# Robotic Assisted Versus Manual Unicompartmental Knee Arthroplasty: A Systematic Review and Meta‐Analysis

**DOI:** 10.1111/os.70424

**Published:** 2026-10-22

**Authors:** Sree Kanakala, Kapil Sugand, Jennifer Tasong, Adrisa Prashar, Harjas Singh Shinmar, Srikar Reddy Namireddy, Saran Singh Gill

**Affiliations:** ^1^ Imperial College London London UK; ^2^ University of Birmingham Birmingham UK; ^3^ Imperial College Healthcare NHS Trust London UK; ^4^ Plovdiv Medical University Plovdiv Bulgaria

**Keywords:** patient reported outcomes, revision rate, robotic surgery, unicompartmental knee arthroplasty

## Abstract

**Background:**

Robotic‐assisted unicompartmental knee arthroplasty (rUKA) aims to enhance the precision and reproducibility of implant positioning compared with manual UKA (mUKA). This systematic review and meta‐analysis compared radiographic, clinical, and patient‐reported outcomes between rUKA and mUKA.

**Methods:**

A systematic search was conducted in PubMed, MEDLINE, EMBASE, Scopus and Web of Science (July 2025) for comparative studies of rUKA and mUKA, in line with the PRISMA statement. Data were pooled using random‐effects models, with effect sizes expressed as mean difference (MD), standardized mean change (SMC), or odds ratio (OR) with 95% confidence intervals (CIs).

**Results:**

Thirty‐four studies encompassing 68,196 UKAs (22,585 robotic; 45,611 manual) were included. rUKA demonstrated significantly lower complication (OR 0.66, 95% CI 0.44–0.99; *p* = 0.045) and revision rates (OR 0.54, 95% CI 0.39–0.75; *p* < 0.001). Operative time was longer for rUKA (+16.1 min; 95% CI 10.8–21.4), while length of stay trended shorter (MD −0.2 days; *p* = 0.44). rUKA was associated with greater functional outcomes, including higher KSS Knee (MD 2.49; *p* = 0.007), KSS Function (MD 2.33; *p* = 0.008), and Oxford Knee Score (SMC 1.06; *p* = 0.049). Radiographically, rUKA yielded lower tibial slope deviation (MD −1.75°; *p* < 0.001) and reduced variability in tibial slope and hip‐knee‐ankle alignment.

**Conclusion:**

rUKA may offer benefit compared to mUKA across radiographic precision, complications, revisions, and functional outcomes, though high heterogeneity in study design and the absence of UKA‐specific minimal clinically important difference thresholds limits the relevance of any rUKA benefit at present. Further large, multicenter, and methodologically robust studies are needed to confirm long‐term results and better define the role of rUKA in clinical practice.

## Graphical abstract

Robotic‐assisted unicompartmental knee arthroplasty is associated with lower complication and revision rates, improved functional outcomes, and more consistent implant positioning compared with manual techniques, albeit with longer operative times. Robotic‐assisted UKA may have a role in contemporary UKA practice.

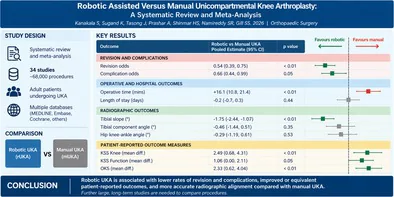

## 1 Introduction

Osteoarthritis (OA) of the knee is a leading cause of pain and disability, affecting approximately 365 million individuals worldwide [1]. In most cases, degeneration is confined to a single tibiofemoral compartment rather than the entire joint [2, 3]. For patients with end stage unicompartmental OA, unicompartmental knee arthroplasty (UKA) offers a bone‐preserving, cruciate‐sparing alternative to total knee arthroplasty (TKA)—the most commonly performed procedure for knee OA. By preserving both cruciate ligaments and resecting only diseased bone, UKA maintains native joint kinematics [4]. Despite these benefits, concerns regarding variability in implant positioning and higher revision rates have limited the widespread adoption of UKA [5]. Consequently, UKA accounts for only 8%–10% of knee arthroplasties in the United Kingdom and United States [6, 7].

UKA is a technically demanding procedure with a steep learning curve [8]. Manual UKA (mUKA) relies heavily on surgical experience and tactile feedback, as restricted visualization can make accurate component placement difficult [9]. Robotic‐assisted UKA (rUKA) has since emerged as a promising adjunct. These systems generate three‐dimensional patient‐specific models to assist planning, while a robotic arm guides bone resection and provides real‐time feedback on joint alignment and soft‐tissue balance. rUKA aims to improve implant positioning, a key determinant of implant survivorship and functional outcomes [10]. Consistent with this, the use of rUKA in the United States increased more than fourfold between 2010 and 2021 [11, 12].

Existing evidence remains divided regarding whether robotic assistance yields better outcomes compared with mUKA [13, 14]. Therefore, this systematic review and meta‐analysis aims to compare the outcomes of rUKA and mUKA. While existing evidence focuses on clinical, radiographic and patient‐reported outcomes separately, we provide a comprehensive synthesis of all three domains [15, 16, 17].

## 2 Methods

### 2.1 Protocol and Registration

This study was prospectively registered with PROSPERO (CRD420251102519) and was conducted in accordance with PRISMA guidelines [18].

### 2.2 Search Strategy, Study Selection and Data Extraction

The literature search was conducted on July 11, 2025 across PubMed, MEDLINE, EMBASE, Scopus and Web of Science databases. The search strategy combined terms relating to knee arthroplasty (“knee arthroplasty” OR “knee replacement” OR “knee”), unicompartmental procedures (“unicompartmental” OR “UKA”) and robotic assistance (“robotic‐assisted” OR “robotic‐arm” OR “robotic” OR “computer‐assisted”) (Table S1). Study selection is detailed in the PRISMA flowchart (Figure 1). The inclusion criteria focused on original, peer‐reviewed, quantitative studies published in English that directly compared a mUKA cohort with a rUKA cohort. Only studies reporting clinical, radiographic, or patient‐reported outcomes (PROM) for both groups were included in the meta‐analysis. All comparative studies meeting these criteria were included in the qualitative synthesis. Studies involving children (< 18 years), reviews, editorials, and case reports were excluded (Table S2). Initial screening and full‐text assessment were performed using Covidence software for duplicate removal and independent review by three reviewers (J.T., A.P., and H.S.S.). Any disagreements were resolved by consensus with SK. Relevant data from each included study were manually extracted on Excel spreadsheets, capturing information on study characteristics (title, authors, country of publication, publication date), surgical factors (implant and robot used), demographic details and study conclusions. This was done by three independent researchers (J.T., A.P., and H.S.S.).

**FIGURE 1:**
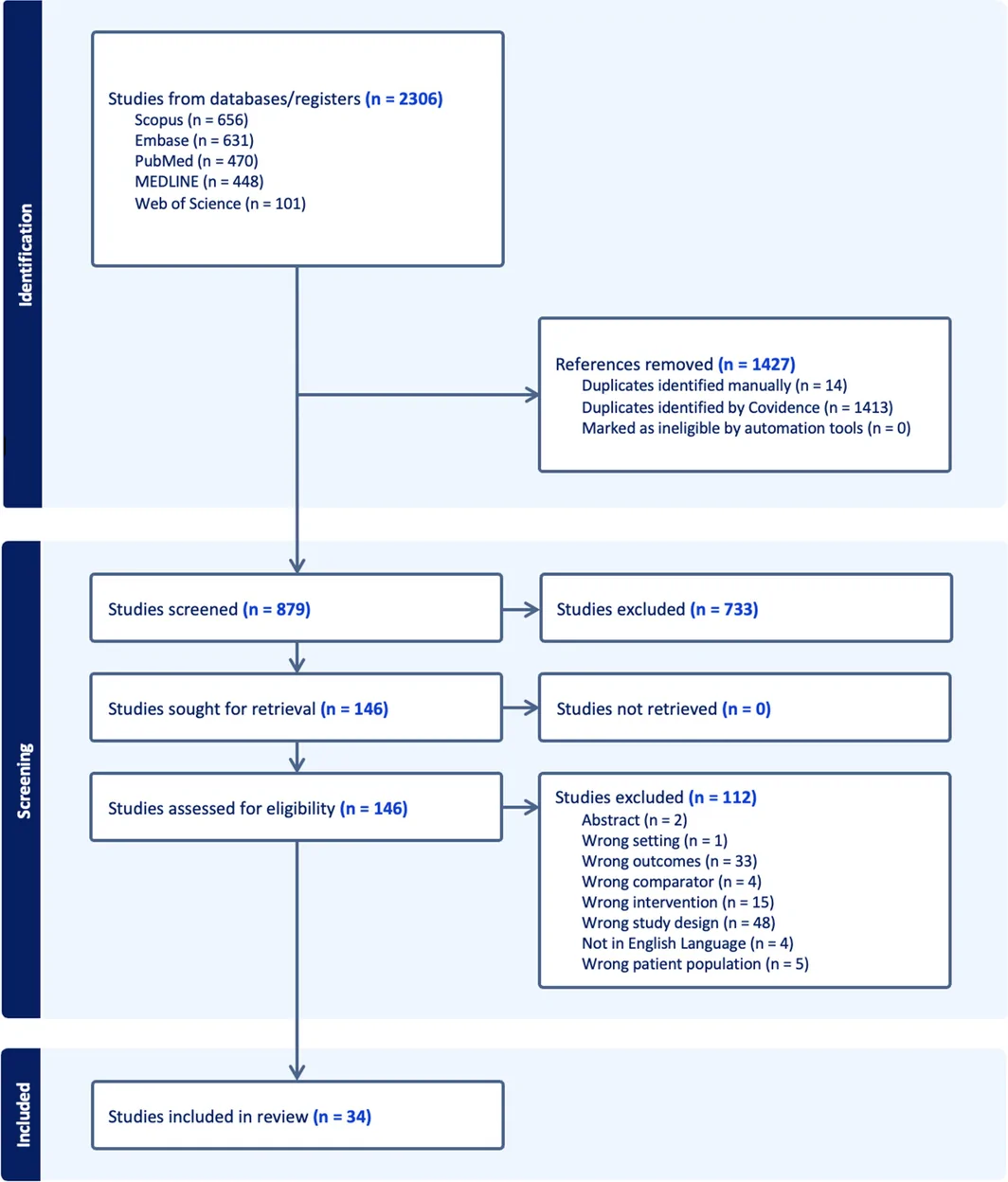
PRISMA flow diagram illustrating the study selection process for the systematic review. A total of 2306 records were identified across five databases (Scopus, Embase, PubMed, MEDLINE, and Web of Science). After removing 1427 duplicates, 879 studies were screened by title and abstract, of which 733 were excluded. The remaining 146 full‐text articles were assessed for eligibility, and 112 were excluded with reasons. Thirty four studies met the inclusion criteria and were included in the qualitative and quantitative synthesis.

### 2.3 Outcomes

The primary outcomes were patient‐reported, radiographic, and perioperative parameters comparing rUKA and mUKA. Patient‐reported outcomes included the Knee Society Score (KSS), Oxford Knee Score (OKS), Western Ontario and McMaster Universities Osteoarthritis Index (WOMAC), and the 12‐Item Short Form Health Survey (SF‐12). Radiographic outcomes included tibial slope angle (TSA), tibial component angle (TCA), and hip‐knee‐ankle angle (HKAA). To quantify implant positioning accuracy, each angle was expressed as the absolute deviation from its optimal postoperative target [15]. Perioperative outcomes included operative time (minutes) and length of stay (days). Complications were defined as any postoperative adverse events documented in the included studies. Revision was defined as any return to theater for exchange or removal of implant components. WOMAC scores were inverted to enable a consistent direction of effect across PROMs. When multiple postoperative timepoints were reported for a measure, the latest available follow‐up was used for meta‐analysis.

### 2.4 Critical Appraisal

Three independent reviewers (J.T., A.P., and H.S.S.) assessed the risk of bias for each study using the Risk of Bias in Non‐Randomized Studies of Interventions (ROBINS‐I) for non‐randomized studies and Risk of Bias 2 (RoB‐2) for randomized studies [19, 20] (Tables S3 and S4). Quality of evidence was assessed using the Oxford Centre for Evidence‐Based Medicine (OCEBM) Levels of Evidence framework [21].

### 2.5 Statistical Analysis

Data preparation was undertaken using Microsoft Excel, and statistical analysis was performed in R (Version 4.4.3) utilizing the *meta* package [22]. Random‐effects models were applied to account for between‐study heterogeneity. Effect sizes were expressed as mean difference (MD) and standardized mean change (SMC) for continuous outcomes and odds ratios (ORs) for binary outcomes, each with 95% confidence intervals (CIs). MD > 0 and SMC > 0 favored rUKA, OR < 1 indicated reduced risk in the rUKA group. For operative time and length of stay, MD > 0 indicated a longer duration for rUKA compared with manual UKA. For radiographic outcomes, MD < 0 favored rUKA. Where appropriate, outcomes were stratified into short (< 12 months), medium (12–36 months) and long (> 36 months) term results. Exploratory subgroup analyses by robot type and study design were not reported because of unstable pooled estimates. Studies with double‐zero events were excluded from quantitative analysis, as they did not influence relative odds ratios between treatment arms [23]. Medians and interquartile ranges were approximated to means and standard deviations [24]. To compare variability in radiographic measures between groups, variance ratios were log‐transformed and converted to standard deviation (SD) ratios. Forest plots were generated to display individual study estimates, 95% confidence intervals (CIs), and pooled estimates. Heterogeneity across studies was quantified using the *I* ^2^ statistic, and publication bias was assessed using funnel plots and Egger's test. Studies were qualitatively synthesized in accordance with SWiM guidelines [25]. Statistical significance was defined at a threshold of *p* < 0.05.

## 3 Results

### 3.1 Study Characteristics

Of 2306 records identified, 1427 duplicates were removed. Eight hundred and seventy‐nine records were screened by title and abstract, of which 733 were excluded. The remaining 146 full‐text articles were assessed for eligibility, and 112 were excluded with reasons (Figure 1). Thirty‐four studies were included in this review [12, 26, 27, 28, 29, 30, 31, 32, 33, 34, 35, 36, 37, 38, 39, 40, 41, 42, 43, 44, 45, 46, 47, 48, 49, 50, 51, 52, 53, 54, 55, 56, 57, 58]. Table 1 summarizes study characteristics. A total of 68,196 UKAs were included, comprising 45,611 manual and 22,585 robotic‐assisted procedures. The pooled mean follow‐up was 23.9 (16.7) months in the manual group and 22.4 (16.9) months in the robotic group. The pooled mean age was 67.7 (9.03) years in the manual group and 68.6 (8.48) years in the robotic group. The pooled BMI was 28.6 (7.55) kg/m^2^ for mUKA and 27.4 (4.11) kg/m^2^ for rUKA. Male patients accounted for 49.3% of the manual cohort and 45.4% of the robotic cohort. The most frequent study design was retrospective cohort (*n* = 17), followed by prospective cohort studies (*n* = 10) and randomized controlled trials (*n* = 7). Table 2 summarizes outcomes for each study.

**TABLE 1:** Summary of included studies comparing robotic‐assisted unicompartmental knee arthroplasty (rUKA) and manual unicompartmental knee arthroplasty (mUKA).

Study	Study type	Level of evidence	Robot	Arm	*n*	% Male	Age (SD)	BMI (SD)	Follow up in months (SD)
Tan 2025	Retrospective Cohort	III	MAKO	rUKA	49	24.2	63.5	27.6	24
				mUKA	110	42.3	63.2	28.1	24
Guild 2025	Retrospective Cohort	III	—	rUKA	3976	47.26	63 (9.2)	NR	24
				mUKA	11,766	47.21	63 (9.1)	NR	24
Rossi 2025	Retrospective Cohort	III	MAKO, NAVIO	rUKA	90	72.3	71.2 (6.1)	27.5 (2.8)	21.7 (11.4)
				mUKA	36	55.6	71.2 (5.4)	28.6 (2.5)	22.3 (12.2)
Sun 2024	RCT	II	China‐made system	rUKA	25	32	67 (4.7)	26.8 (2.3)	3
				mUKA	25	24	66.3 (3.8)	27.3 (1.4)	3
Lim 2025	Retrospective Cohort	III	MAKO	rUKA	64	52	62.4 (9.4)	28.7 (4.7)	12
				mUKA	192	55	63.2 (8.4)	28.7 (4.4)	12
Lau 2024	Retrospective Case–Control	III	NAVIO	rUKA	56	20.7	69.6 (7.3)	25.9 (3.4)	36
				mUKA	82	35.4	70.9 (7.9)	26.4 (3.7)	36
Kwon 2025	Retrospective Cohort	III	MAKO	rUKA	35	74.3	65.1 (6.7)	27.1 (3)	12
				mUKA	35	71.4	63.3 (5)	26.5 (2.7)	12
Yeung 2023	Retrospective Case–Control	III	MAKO	rUKA	74	NR	NR	NR	18.1 (6.1)
				mUKA	74	NR	NR	NR	18.1 (6.1)
Foissey 2023	Retrospective Cohort	III	NAVIO	rUKA	197	45.2	66.7 (7.7)	27.5 (3.3)	49.4 (13.4)
				mUKA	159	48.4	68.3 (8.1)	27 (3.4)	76.1 (30.3)
Maritan 2023	Retrospective Cohort	III	MAKO	rUKA	52	21.2	60.9 (8.4)	26.2 (3.3)	90.4 (9.1)
				mUKA	43	14	61.5 (8.5)	27 (2.8)	95.4 (11)
Crizer 2021	Retrospective Cohort	III	NAVIO	rUKA	50	58	63 (11)	28.1 (4.45)	24
				mUKA	39	56.4	58 (13)	28.3 (4.06)	24
Banger 2021	RCT	II	MAKO	rUKA	55	NR	NR	NR	60
				mUKA	49	NR	NR	NR	60
Negrin 2021	Prospective Cohort	II	NAVIO	rUKA	16	44	66 (6.5)	NR	NR
				mUKA	18	67	65 (8.75)	NR	NR
Thilak 2020	Prospective Cohort	II	MAKO	rUKA	24	NR	57.5 (5)	NR	NR
				mUKA	12	NR	53.7 (5.1)	NR	NR
Mergenthaler 2021	Retrospective Case–Control	III	NAVIO	rUKA	200	39	66.7 (9.3)	27 (4.2)	22.5 (12.9)
				mUKA	191	30.4	67.1 (10.7)	26.4 (4.2)	30.2 (23.4)
St Mart 2020	Retrospective Cohort	III	MAKO	rUKA	2851	56.4	65.7 (9.3)	NR	16.8 (10.68)
				mUKA	3093	58.9	65.4 (9.9)	NR	21.6 (13.44)
Park 2019	Retrospective Cohort	III	MAKO	rUKA	55	20	64.8 (3.25)	25.5 (2.5)	27.8
				mUKA	57	12.2	68.4 (3.5)	25.9 (3.7)	27.8
Vakharia 2021	Retrospective Cohort	III	—	rUKA	13,617	42.55	71.2 (6.8)	NR	NR
				mUKA	21,444	47.61	71.6 (6.6)	NR	NR
Cool 2019	Retrospective Cohort	III	—	rUKA	246	46.3	NR	NR	NR
				mUKA	492	42.7	NR	NR	NR
Wong 2019	Retrospective Cohort	III	MAKO	rUKA	58	51	70.4 (9.7)	28.2 (5.6)	24
				mUKA	118	37	67.9 (9.5)	28.7 (4.4)	24
Kayani 2019	Prospective Cohort	II	MAKO	rUKA	73	43.8	65.3 (8.6)	28.7 (4.1)	24
				mUKA	73	46.6	66.1 (5.8)	27.9 (2.3)	24
Canetti 2018	Retrospective Cohort	III	NAVIO	rUKA	11	18	66.5 (6.8)	24.2 (4.3)	34.3 (10.5)
				mUKA	17	28	59.5 (9.9)	26.3 (3.8)	39.3 (15.5)
Batailler 2019	Retrospective Case–Control	III	NAVIO	rUKA	80	33.8	69 (9.6)	26.1 (4.1)	19.7 (9)
				mUKA	80	33.8	68 (10)	25.5 (3.9)	24.2 (16)
Gilmour 2018	RCT	II	MAKO	rUKA	58	55	61.8 (7.84)	NR	24
				mUKA	54	52	62.6 (7.13)	NR	24
Blyth 2017	RCT	II	MAKO	rUKA	64	53.9	62.5 (6.9)	NR	12
				mUKA	62	56.3	61.7 (7.9)	NR	12
MacCallum 2016	Retrospective Cohort	III	MAKO	rUKA	87	NR	NR	NR	32.4
				mUKA	177	NR	NR	NR	32.4
Hansen 2014	Retrospective Cohort	III	MAKO	rUKA	30	53.30	63.1 (9.8)	32.13 (5.49)	24
				mUKA	32	34.40	60.66 (11.78)	31.3 (5.7)	24
Lonner 2010	Prospective Cohort	II	MAKO	rUKA	31	48.39	64	30 (5)	1.5
				mUKA	27	63	57	28 (4)	1.5
Cobb 2006	RCT	II	ACROBOT	rUKA	15	53.57	69.8 (5.7)	NR	4.5
				mUKA	13	53.57	70.4 (5.1)	NR	4.5
Strait 2024	Prospective Cohort	II	MAKO	rUKA	85	69.4	61.85 (8.21)	29.14	24
				mUKA	312	44.9	61.36 (9.82)	30	24
Blyth 2025	RCT	II	MAKO	rUKA	75	48	67	28.7	60
				mUKA	75	48	67	28.7	60
Wu 2022	Prospective Cohort	II	MAKO	rUKA	52	21.2	68.52 (9.78)	NR	24
				mUKA	61	14.8	60.36 (9.14)	NR	24
Kayani 2018	Prospective Cohort	II	MAKO	rUKA	74	50	67	28.5	24
				mUKA	65	50	67	28.5	24
Bell 2016	RCT	II	MAKO	rUKA	62	53.9	62.5 (6.9)	NR	3
				mUKA	58	56.3	61.7 (7.9)	NR	3

*Note:* Study design, level of evidence, robotic system used, sample size, demographic characteristics, and follow‐up duration are presented for each study arm. Follow‐up is reported in months. The majority of studies were retrospective cohort designs (Level III evidence), with a minority being randomized controlled trials or prospective cohorts (Level II). MAKO was the most frequently employed robotic system, followed by NAVIO and other image‐free or region‐specific platforms. Follow‐up periods ranged from 1.5 to 95 months across studies.

**TABLE 2:** Summary of perioperative, radiographic, and patient‐reported outcomes at the most recent postoperative follow‐up reported for each study arm.

Study	Arm	Length of stay (days)	Operative time (minutes)	Complication rate (%)	Revision rate (%)	TSA (°)	TCA (°)	HKAA (°)	KSS Knee	KSS function	OKS	SF‐12 MCS	SF‐12 PCS	WOMAC
Banger 2021	mUKA	—	—	12.2	4.1	—	—	—	88.0 (3.6)	88.0 (3.6)	41.0 (2.3)	—	—	—
	rUKA	—	—	0	0	—	—	—	89.0 (4.8)	89.0 (4.3)	40.0 (2.2)	—	—	—
Batailler 2019	mUKA	—	—	7.5	18	5.8 (5.8)		4.7 (175.3)	87.7 (15.0)	92.0 (13.0)		—	—	—
	rUKA	—	—	5	5	3.6 (3.6)		4.8 (175.2)	90.0 (11.0)	92.6 (13.0)		—	—	—
Bell 2016	mUKA	—	—	—	—	3.7 (3.7)	2.7 (2.7)	—	—	—	—	—	—	—
	rUKA	—	—	—	—	1.0 (1.0)	1.6 (1.6)	—	—	—	—	—	—	—
Blyth 2017	mUKA	—	—	22	0	—	—	—	—	—	38.7 (8.2)	54.6 (8.3)	44.6 (9.8)	—
	rUKA	—	—	7	0	—	—	—	—	—	38.7 (8.9)	54.9 (8.3)	46.8 (9.8)	—
Blyth 2025	mUKA	—	80.0 (18.0)	4	—	—	—	—	—	—	—	—	—	—
	rUKA	—	89.0 (20.0)	0	0	—	—	—	—	—	—	—	—	—
Canetti 2018	mUKA	—	—	5.9	—	—	—	—	91.2 (6.5)	97.1 (9.9)	—	—	—	—
	rUKA	—	—	0	—	—	—	—	97.2 (5.9)	96.4 (9.2)	—	—	—	—
Cobb 2006	mUKA	—	88.0 (16.0)	20	—	—	—	—	—	—	—	—	—	—
	rUKA	—	104.0 (17.0)	30.8	—	—	—	—	—	—	—	—	—	—
Cool 2019	mUKA	2.0 (0.8)	—	—	5.28	—	—	—	—	—	—	—	—	—
	rUKA	1.8 (0.7)	—	—	0.81	—	—	—	—	—	—	—	—	—
Crizer 2021	mUKA	—	—	—	0	—	—	—	56.0 (17.0)	74.0 (19.0)	—	51.0 (7.0)	48.0 (10.0)	—
	rUKA	—	—	—	0	—	—	—	66.0 (17.0)	82.0 (17.0)		55.0 (7.0)	50.0 (11.0)	—
Foissey 2023	mUKA	—	—	—	12.6	7.0 (7.0)	4.1 (4.1)	3.4 (176.6)	86.3 (12.1)	86.5 (13.8)	—	—	—	—
	rUKA	—	—	—	3	4.7 (4.7)	3.9 (3.9)	3.5 (176.5)	91.5 (7.3)	91.7 (8.3)	—	—	—	—
Gilmour 2018	mUKA	—	—	67	—	—	—	—	—	—	39.9 (6.1)	—	—	—
	rUKA	—	—	29.3	0	—	—	—	—	—	38.9 (9.0)	—	—	—
Guild 2025	mUKA	—	—	9.41	2.74	—	—	—	—	—	—	—	—	—
	rUKA	—	—	8.78	2.14	—	—	—	—	—	—	—	—	—
Hansen 2014	mUKA	—	88.8 (21.0)	9.4	0	8.3 (8.3)	1.1 (1.1)	—	—	—	—	—	—	—
	rUKA	—	100.8 (16.8)	23.3	0	6.1 (6.1)	1.6 (1.6)	—	—	—	—	—	—	—
Kayani 2018	mUKA	—	82.0 (18.0)	3.1	—	—	—	—	—	—	—	—	—	—
	rUKA	—	89.0 (19.0)	0	—	—	—	—	—	—	—	—	—	—
Kayani 2019	mUKA	3.0 (0.6)	—	3.33	—	—	—	—	—	—	—	—	—	—
	rUKA	1.8 (0.2)	—	0	—	—	—	—	—	—	—	—	—	—
Kwon 2025	mUKA	—	—	—	—	—	—	0.0 (180.0)	90.1 (7.2)	78.8 (8.5)	—	—	—	18.4 (7.6)
	rUKA	—	—	—	—	—	—	1.5 (178.5)	92.2 (8.6)	81.6 (9.0)	—	—	—	12.9 (4.9)
Lau 2024	mUKA	—	101.0 (14.0)	—	3	—	—	5.0 (175.0)	—	—	—	—	—	—
	rUKA	—	119.0 (16.5)	—	1	—	—	5.0 (175.0)	—	—	—	—	—	—
Lim 2025	mUKA	0.9 (0.9)	—	5.7	1	—	—	—	—	—	—	—	—	—
	rUKA	0.8 (0.7)	—	3.1	0	—	—	—	—	—	—	—	—	—
Lonner 2010	mUKA	—	—	0	0	—	—	—	—	—	—	—	—	—
	rUKA	—	—	3.2	3.4	—	—	—	—	—	—	—	—	—
MacCallum 2016	mUKA	—	87.1 (15.0)	—	4	4.9 (4.9)	3.9 (3.9)	—	—	—	—	—	—	—
	rUKA	—	103.7 (19.7)	—	3.4	2.4 (2.4)	2.6 (2.6)	—	—	—	—	—	—	—
Maritan 2023	mUKA	—	—	7	7	—	—	—	—	—	—	—	—	—
	rUKA	—	—	3.8	3.8	—	—	—	—	—	—	—	—	—
Mergenthaler 2021	mUKA	—	76.0 (21.0)	6.8	11	—	—	—	87.3 (13.7)	88.4 (16.1)	—	—	—	—
	rUKA	—	81.0 (21.0)	5.7	4	—	—	—	91.9 (10.6)	92.8 (13.4)	—	—	—	—
Negrin 2021	mUKA	2.0 (0.5)	107.0 (11.5)	5.6	—	—	—	—	—	—	39.0 (1.2)	—	—	—
	rUKA	2.3 (0.8)	139.8 (7.8)	0	0	—	—	—	—	—	45.0 (1.5)	—	—	—
Park 2019	mUKA	—	—	—	—	—	2.3 (2.3)	2.1 (177.9)	87.8 (8.3)	70.8 (13.1)	—	—	—	27.8 (16.5)
	rUKA	—	—	—	—	—	0.4 (0.4)	1.2 (178.8)	83.6 (18.6)	68.4 (16.0)	—	—	—	29.9 (18.1)
Rossi 2025	mUKA	3.2 (1.7)	86.9 (12.0)	0	2.8	5.0 (5.0)		3.6 (176.4)	88.9 (14.3)	87.5 (16.4)	—	—	—	—
	rUKA	3.8 (1.4)	115.0 (22.0)	1.1	2.2	2.7 (2.7)		3.7 (176.3)	91.7 (11.9)	91.9 (11.9)	—	—	—	—
St Mart 2020	mUKA	—	—	—	3.7	—	—	—	—	—	—	—	—	—
	rUKA	—	—	—	2.6	—	—	—	—	—	—	—	—	—
Strait 2024	mUKA	—	73.0 (14.0)	0.9	0.32	—	—	—	—	—	32.9 (10.0)	51.6 (9.2)	39.5 (12.2)	—
	rUKA	—	88.0 (15.0)	0	0	—	—	—	—	—	43.5 (4.9)	59.3 (3.4)	51.1 (5.6)	—
Sun 2024	mUKA		75.3 (10.2)	—	—	—	3.2 (3.2)	—	87.7 (9.1)	80.4 (6.9)	—	—	—	—
	rUKA		93.5 (8.5)	—	—	—	2.2 (2.2)	—	88.8 (7.3)	81.3 (7.4)	—	—	—	—
Tan 2025	mUKA	4.3 (1.4)	92.2 (19.6)	2.73	—	5.5 (5.5)	—	—	—	—	—	—	—	—
	rUKA	3.9 (2.0)	127.4 (36.7)	2.04	—	5.4 (5.4)	—	—	—	—	—	—	—	—
Thilak 2020	mUKA	—	—	—	—	7.4 (7.4)	—	—	—	—	—	—	—	—
	rUKA	—	—	—	—	7.1 (7.1)	—	—	—	—	—	—	—	—
Vakharia 2021	mUKA	—	—	—	4.24	—	—	—	—	—	—	—	—	—
	rUKA	—	—	—	0.99	—	—	—	—	—	—	—	—	—
Wong 2019	mUKA	—	88.6 (11.3)	6.6	6.8	—	—	—	—	77.7 (21.3)		53.1 (8.7)	45.7 (11.2)	19.9 (18.2)
	rUKA	—	109.1 (19.2)	12.1	12	—	—	—	—	83.4 (14.7)		53.8 (7.3)	43.9 (9.5)	16.4 (14.3)
Wu 2022	mUKA	—	43.9 (6.1)	0	0	10.0 (10.0)	2.4 (2.4)	5.9 (174.1)	—	—	—	—	—	10.5 (11.4)
	rUKA	—	60.9 (11.4)	1.9	0	10.4 (10.4)	4.9 (4.9)	2.8 (177.2)	—	—	—	—	—	14.7 (11.1)
Yeung 2023	mUKA	—	101.4 (36.8)	—	4.1	8.2 (8.2)	1.7 (1.7)	4.8 (175.2)	93.1 (8.0)	79.0 (36.5)	—	—	—	—
	rUKA	—	93.0 (20.6)	—	4.1	5.7 (5.7)	‐0.3 (−0.3)	4.4 (175.6)	92.2 (5.6)	71.5 (14.7)	—	—	—	—

*Note:* Data are presented separately for manual and robotic unicompartmental knee arthroplasty arms. Length of stay and operative time are reported as mean values with standard deviations in parentheses. Complication and revision rates are reported as percentages. All radiographic angles, including tibial slope angle (TSA), tibial component angle (TCA), and hip‐knee‐ankle angle (HKAA), are normalized to postoperative reference values. Patient‐reported outcomes include the Knee Society Score (KSS) knee and function subscales, Oxford Knee Score (OKS), SF‐12 Mental Component Summary (MCS), SF‐12 Physical Component Summary (PCS), and Western Ontario and McMaster Universities Osteoarthritis Index (WOMAC).

### 3.2 Implant and Robot Types

In rUKA studies, the MAKO system was the most frequently reported robotic platform (*n* = 22), followed by NAVIO (*n* = 8), ACROBOT (*n* = 1), and a China‐manufactured robotic system (*n* = 1). Two studies reported on multiple robotic systems, and two studies did not specify type of robotic system. In mUKA studies, the Oxford Phase 3 implant was the most frequently reported (*n* = 14), followed by Zimmer unicompartmental systems (*n* = 10), Journey UNI (*n* = 6), and HLS Uni Evolution implant (*n* = 4).

### 3.3 Risk of Bias and Publication Bias

Of the RCTs assessed, two were judged to be at low risk of bias and five were rated as having some concerns (Figure S1). This was primarily due to deviations from the intended intervention and insufficient reporting of randomization procedures. None was judged to be at high risk. Among non‐randomized studies, the majority were rated at moderate risk of bias (*n* = 15) (Figure S2). This was due to potential confounding and selection bias inherent to observational designs. Seven studies demonstrated low risk of bias, while five were judged to be at serious risk due to lack of adjustment for baseline differences. All studies classified the intervention appropriately and few deviated from the intended intervention. No studies were deemed to be at critical risk. Funnel plots for tibial slope angle, tibial component angle, and operative time demonstrated mild visual asymmetry (Figures S3–S9). Plots for other outcomes appeared symmetrical. Egger's test for all outcomes did not demonstrate statistical significance (*p* > 0.05) (Table S5).

### 3.4 Perioperative Outcomes

Fifteen studies compared operative time between arms, including 984 rUKA and 1394 mUKA patients. Operative times ranged from 43.9 min [56] to 180 min [47]. All studies bar one reported a longer operative time for robotic compared to manual UKA [32]. Definition of operative time varied across studies. Six studies compared length of stay between arms, including 538 rUKA and 921 mUKA patients. Reported stays ranged from 0.8 days [29] to 4.3 days [26]. Four studies found the robotic approach to result in a shorter stay.

### 3.5 Complication Rate

Twenty‐one studies, including 5206 robotic and 13,396 manual UKAs, reported postoperative complications. Complication rates ranged from 0% [56] to 14.3% [46] with a median rate of 3.1% (IQR 0, 7%) for robotic and 5.9% (IQR 3.1, 9.4%) for manual cohorts. The lowest rates were reported in recent high‐volume robotic series [36]. Earlier manual series demonstrated higher complication frequencies [52]. Guild et al., the largest study in this analysis (*n* = 15,472), found no significant difference in overall complication rate between robotic and manual arms [12].

Across studies, the most common complications were superficial wound infection (*n* = 14/21 studies), deep vein thrombosis (*n* = 12/21 studies), and stiffness requiring manipulation under anesthesia (*n* = 10/21 studies). Less frequent events included aseptic loosening, progression of osteoarthritis in the unreplaced compartment, and bearing dislocation. Major complications such as deep infection, periprosthetic fracture, and neurovascular injury occurred in < 1% of cases. Definitions of complication and follow‐up duration varied between studies (Table S6).

### 3.6 Revision Rate

Eighteen studies, including 8202 robotic and 20,266 manual UKAs, reported revision rates. Reported rates ranged from 0% [36] to 18.0% [47], with a median rate of 1.6% (IQR 0.0, 3.4%) for robotic and 2.9% (IQR 0.0, 5.3%) for manual cohorts. Guild et al., the largest study in this analysis with 15,742 patients, found rUKA to show a significantly lower aseptic revision rate than mUKA (*p* = 0.04) [12].

### 3.7 Patient Reported Outcome Measures

KSS was the most frequently reported measure (*n* = 24), followed by OKS (*n* = 10), WOMAC (*n* = 8), and SF‐12 (*n* = 8). Short‐term follow‐up showed largely equivalent postoperative improvements between groups [29, 56]. At longer‐term follow up, robotic UKA exceeded manual UKA in some PROMs [33, 34].

### 3.8 Radiographic Measures

Tibial component angle was reported in eight studies, including 582 robotic and 643 manual UKAs. Tibial slope was reported in 10 studies, including 745 robotic and 799 manual UKAs. Hip‐knee‐ankle angle was reported in eight studies, including 639 robotic and 584 manual UKAs. Across all parameters, both approaches achieved alignment within accepted target ranges. Studies frequently found better accuracy and lower alignment variability in the robotic group [33, 57].

## 4 Meta‐Analysis

### 4.1 Complication Rate

Complication rates were analyzed from 21 studies, including 5206 robotic and 13,396 manual UKAs. The median follow‐up for both rUKA and mUKA was 24 months (rUKA IQR: 17.8–24.0, mUKA IQR: 19.7–25.7). Robotic UKA demonstrated a significantly lower complication rate compared with manual UKA, with a pooled odds ratio of 0.66 (95% CI: 0.44–0.99, *I* ^2^ = 39.6%, *p* = 0.045) (Figure 2). Similar results were shown in timepoint stratified analyses (Figure S10).

**FIGURE 2:**
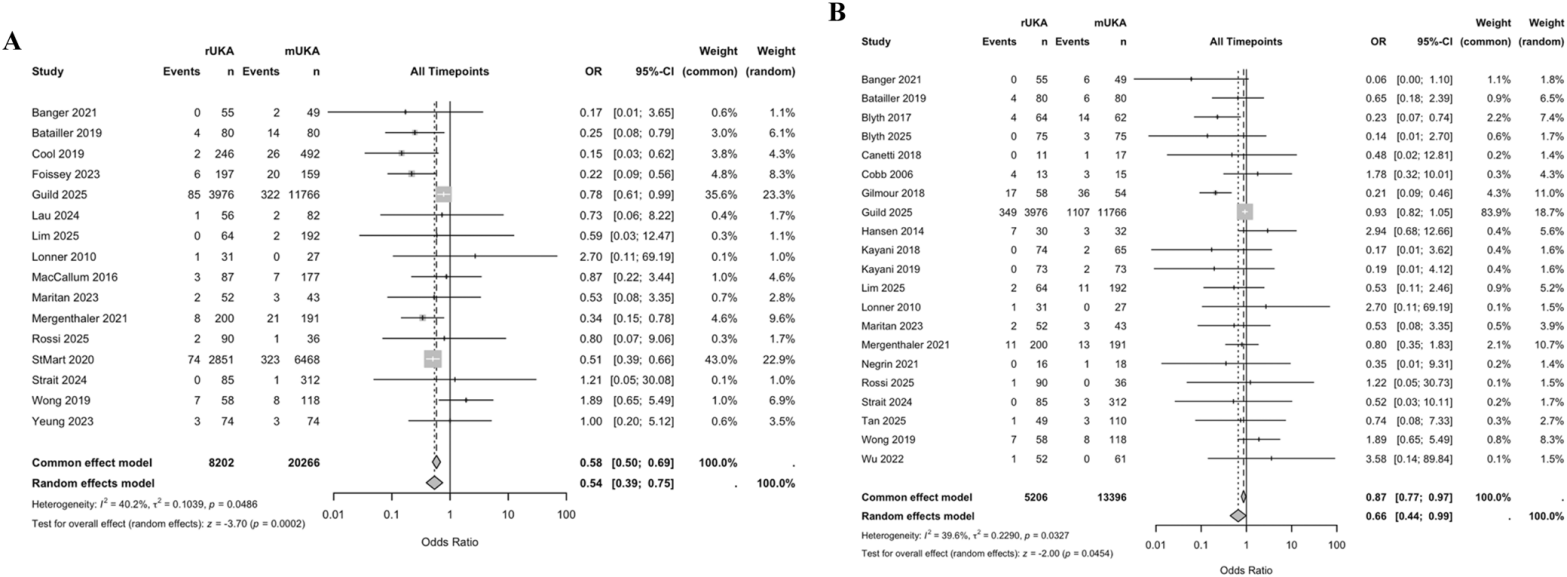
Forest plots comparing complication and revision rates between robotic‐assisted unicompartmental knee arthroplasty (rUKA) and manual unicompartmental knee arthroplasty (mUKA). (A) Revision rate: The pooled odds ratio (OR) for revision was 0.54 [0.39, 0.75], favoring rUKA, with moderate heterogeneity (*I* ^2^ = 40.2%, *p* = 0.0486). (B) Complication rate: The pooled OR for complications was 0.66 [0.44, 0.99], again favoring rUKA, with moderate heterogeneity (*I* ^2^ = 39.6%, *p* = 0.0327). In both analyses, individual and pooled effect estimates are displayed with corresponding 95% confidence intervals (CIs). Squares represent individual study weights, and diamonds indicate pooled estimates under a random‐effects model.

### 4.2 Revision Rate

Revision rates were analyzed from 16 studies, including 8202 robotic and 20,266 manual UKAs. The median follow‐up for both rUKA and mUKA was 24 months (rUKA IQR: 20.2–30.3, mUKA IQR: 23.0–30.8). Robotic UKA was associated with a significantly lower revision rate compared with manual UKA, with a pooled odds ratio of 0.54 (95% CI: 0.39–0.75, *I* ^2^ = 40.2%, *p* < 0.001) (Figure 2). Similar results were shown in timepoint stratified analyses (Figure S10).

### 4.3 Perioperative Outcomes

Operative time was analyzed from 15 studies, including 984 robotic and 1391 manual UKAs. Robotic assisted procedures were associated with a significantly longer operative duration compared with manual UKA (MD: +16.1 min, 95% CI: 10.8–21.4, *I* ^2^ = 88.6%). Length of stay was analyzed from five studies, including 538 robotic and 921 manual UKAs. There was no statistically significant difference between groups, though the mean difference favored rUKA (MD: −0.2 days, 95% CI: −0.7 to 0.3, *I* ^2^ = 96.4%, *p* = 0.44) (Figure 3).

**FIGURE 3:**
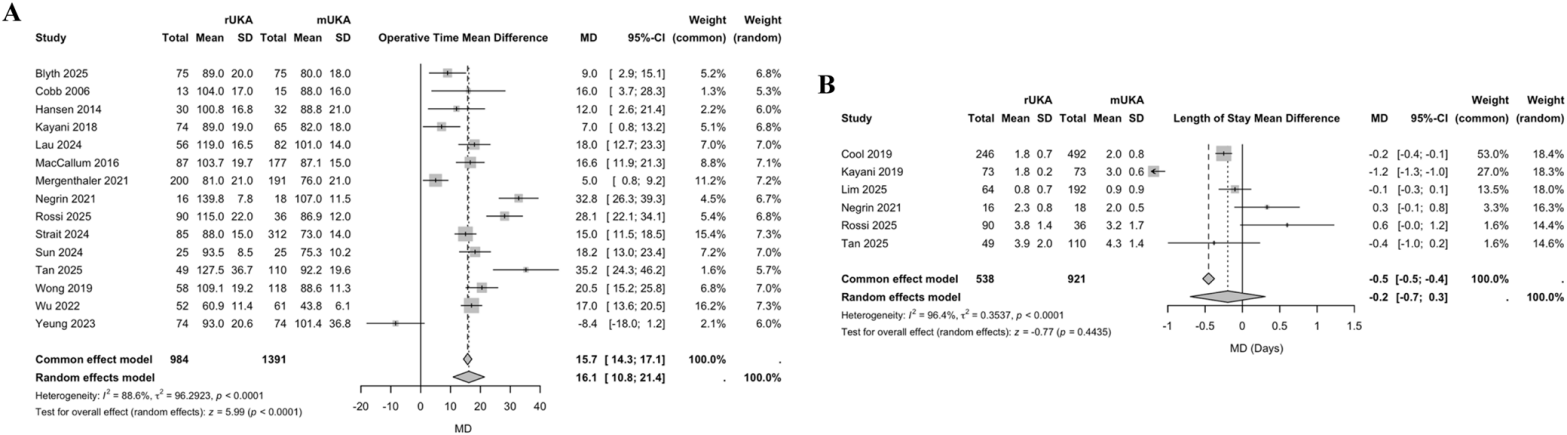
Forest plots comparing perioperative outcomes between robotic‐assisted unicompartmental knee arthroplasty (rUKA) and manual unicompartmental knee arthroplasty (mUKA). (A) Operative time: The pooled mean difference (MD) for operative duration was 16.1 min [10.8, 21.4], indicating significantly longer procedures with rUKA, with very high heterogeneity (*I* ^2^ = 88.6%, *p* < 0.0001). (B) Length of stay: The pooled MD for hospital stay was −0.2 days [−0.7, 0.3], showing no significant difference between rUKA and mUKA, despite substantial heterogeneity (*I* ^2^ = 98.4%, *p* < 0.0001). In both analyses, individual and pooled effect estimates are presented with their corresponding 95% confidence intervals (CIs). Squares represent individual study weights, and diamonds indicate pooled estimates under a random‐effects model.

### 4.4 Radiographic Measures

Tibial slope was analyzed from 10 studies, including 745 robotic and 799 manual UKAs. Robotic UKA was associated with a lower absolute deviation from the postoperative target compared to manual UKA (MD: −1.75°; 95% CI: −2.44–(−)1.07; *p* < 0.001). Tibial component angle was analyzed from eight studies, including 582 robotic and 643 manual UKAs. Hip‐knee‐ankle angle was analyzed from eight studies, including 639 robotic and 584 manual UKAs. TCA and HKAA favored rUKA, although trends did not reach statistical significance. Robotic UKA showed significantly lower variability in postoperative tibial slope (SD ratio = 0.68, 95% CI 0.54–0.84; *p* < 0.001) and hip‐knee‐ankle angle (SD ratio = 0.78, 95% CI 0.66–0.92; *p* = 0.004). Variability in tibial component angle favored robotic UKA but did not reach statistical significance (Figure 4).

**FIGURE 4:**
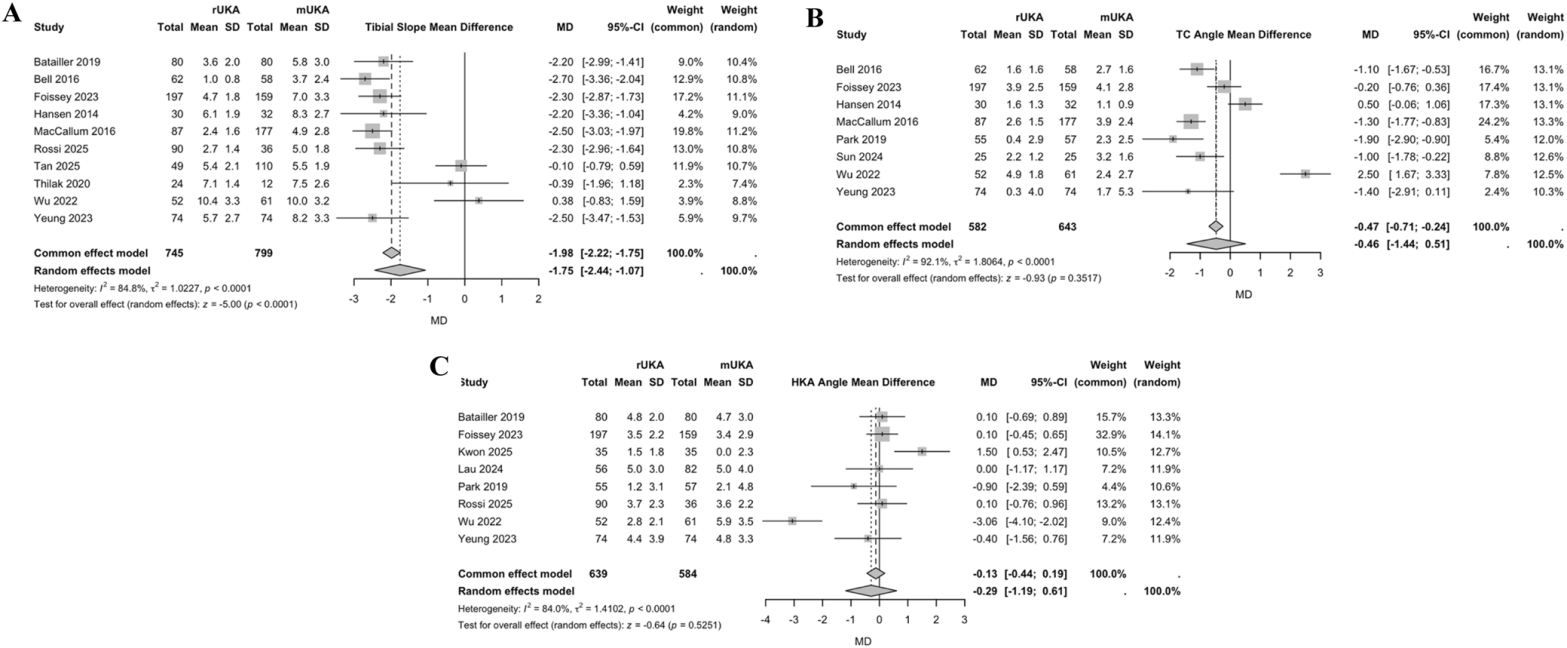
Forest plots comparing radiographic alignment outcomes between robotic‐assisted unicompartmental knee arthroplasty (rUKA) and manual unicompartmental knee arthroplasty (mUKA). (A) Tibial slope angle: The pooled mean difference (MD) was −1.75° [−2.44, −1.07], favoring rUKA for achieving tibial slope values closer to the postoperative target, with high heterogeneity (*I* ^2^ = 84.8%, *p* < 0.0001). (B) Tibial component angle: The pooled MD was −0.46° [−1.44, 0.51], showing no significant difference between rUKA and mUKA, with very high heterogeneity (*I* ^2^ = 92.1%, *p* < 0.0001). (C) Hip‐knee‐ankle angle: The pooled MD was −0.29° [−1.41, 0.84], indicating no significant difference in overall limb alignment between rUKA and mUKA, with high heterogeneity (*I* ^2^ = 84.0%, *p* < 0.0001). In all analyses, individual and pooled mean differences are shown with corresponding 95% confidence intervals (CIs). Squares represent individual study weights, and diamonds indicate pooled estimates under a random‐effects model.

### 4.5 Patient Reported Outcome Measures

The median follow‐up for studies reporting PROMs was 24 months (rUKA IQR: 19.3–24.9, mUKA IQR: 21.2–28.4). Robotic UKA was associated with significantly higher postoperative KSS Function (MD = 2.33; 95% CI 0.62–4.04; *p* = 0.008) and KSS Knee (MD = 2.49; 95% CI 0.68–4.31; *p* = 0.007) compared with manual UKA. Robotic UKA demonstrated significantly greater improvement in Oxford Knee Score (SMC = 1.06; 95% CI 0.00–2.11; *p* = 0.049) than manual UKA. All remaining PROMs demonstrated a trend favoring robotic UKA, though they did not reach statistical significance (Figure 5). Similar results were shown in timepoint stratified analyses (Figure S11).

**FIGURE 5:**
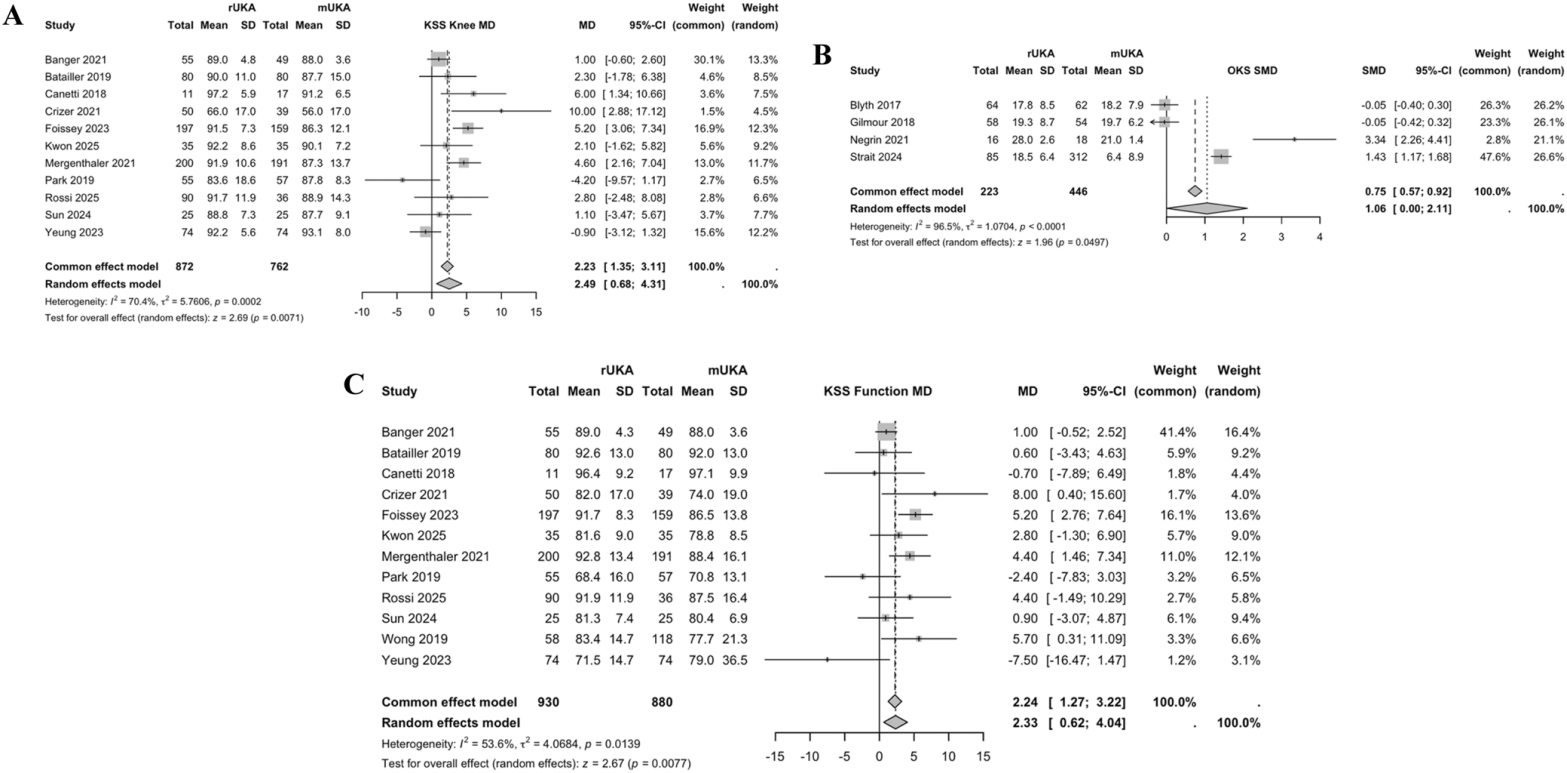
Forest plots comparing patient‐reported outcome measures between robotic‐assisted unicompartmental knee arthroplasty (rUKA) and manual unicompartmental knee arthroplasty (mUKA). (A) Knee Society Score (KSS) Knee: The pooled mean difference (MD) was 2.49 [0.68, 4.31], favoring rUKA, with moderate heterogeneity (*I* ^2^ = 70.4%, *p* = 0.0002). (B) Oxford Knee Score (OKS): The pooled standardized mean change (SMC) was 1.06 [0.00, 2.11], also favoring rUKA, with substantial heterogeneity (*I* ^2^ = 86.5%, *p* < 0.0001). (C) Knee Society Score (KSS) Function: The pooled MD was 2.33 [0.62, 4.04], again favoring rUKA, with moderate heterogeneity (*I* ^2^ = 53.6%, *p* = 0.0139). In all analyses, individual and pooled effect estimates are presented with their corresponding 95% confidence intervals (CIs). Squares represent individual study weights, and diamonds indicate pooled estimates under a random‐effects model.

## 5 Discussion

### 5.1 Review Summary

This systematic review and meta‐analysis, encompassing 68,000 procedures from 34 studies, represents a comprehensive synthesis of outcomes comparing robotic and manual UKA. Our analysis provides evidence that rUKA may be associated with improved outcomes when compared to manual UKA, with statistically significantly lower rates of complications and revision, alongside superior or equivalently improving outcomes in both PROMs and radiographic alignment.

### 5.2 Patient‐Reported Outcomes

While earlier meta‐analyses have reported mixed results, they are limited by smaller sample sizes and older studies [15, 17]. Our findings demonstrate that rUKA is associated with significantly greater postoperative PROMs and greater improvement from baseline, suggesting that it may better restore function and patient satisfaction compared with mUKA. However, interpretation is limited by the absence of minimum clinically important difference (MCID) thresholds validated specifically for UKA. Existing MCIDs are derived from TKA cohorts, with reported values of 5.3–5.9 for KSS Knee and 6.1–6.4 for Function [59]. It is plausible that UKA, as a less invasive procedure typically offered to higher‐functioning patients, may have different MCID values, but this remains unproven. Until UKA‐specific thresholds are established, the true clinical significance of PROM improvements with rUKA cannot be determined. Nonetheless, emerging evidence suggests rUKA may support faster recovery and earlier return to function [60], though the differences in PROMs between rUKA and mUKA observed remain below current TKA MCIDs. Future work should establish UKA‐specific MCIDs.

### 5.3 Radiographic Outcomes

Improved radiographic precision is central to the theoretical advantage of rUKA [60]. In line with the broader literature, our analysis found that both robotic and manual techniques achieved satisfactory alignment within accepted target ranges [15]. While existing meta‐analyses found no significant differences in TS, TCA, or HKAA, the present analysis shows a statistically significant improvement in tibial slope alignment favoring robotic assistance [15]. This finding was likely influenced by the large contribution of Rossi et al. 2025, a retrospective cohort study of 126 knees [27]. The study reported significantly lower tibial slope deviation and improved joint‐line restoration in the robotic group [61]. However, the imbalance in group sizes (rUKA: *n* = 90, mUKA: *n* = 36) may have biased the pooled estimate toward a greater apparent advantage for robotic assistance. Alignment strategy is surgeon specific, and by applying uniform target thresholds, our analysis may not fully capture this nuance. Nonetheless, the lower variability in radiographic angles observed with rUKA suggests that, irrespective of alignment approach, robotic assistance may improve reproducibility and reduce outliers in component placement, compared with mUKA. These differences may help explain the lower revision and complications observed with rUKA, though the clinical significance of radiographic precision remains uncertain. Correlations of radiographic angles with PROMs and implant survival in the UKA population are needed [60].

### 5.4 Complications and Revisions

Complication and revision rates are important indicators of procedural safety and long‐term implant success. In our analysis, the overall effect was largely driven by several large registry‐based and high‐volume cohort studies, most notably Guild et al.'s propensity score‐matched retrospective cohort of UKA and rUKA performed between 2010 and 2021 [12]. At both 1 and 2 years postoperatively, the rUKA cohort showed significantly lower rates of aseptic revision, aseptic loosening, and all‐cause reoperation, while rates of 90‐day medical complications were comparable between groups. Registry data such as these provide long‐term longitudinal follow‐up and allow generalizability to real‐world practice. Our effect estimates suggest that rUKA may be associated with significantly lower revision and complication rates compared to mUKA, though results should be interpreted with caution due to high heterogeneity and different follow‐up periods. The mechanism underlying this signal favoring rUKA is complex and poorly defined in the existing literature. Greater precision in component alignment and soft‐tissue balance may reduce complications such as aseptic loosening and progression of osteoarthritis in the unreplaced compartment [60]. Furthermore, robotic assistance may help standardize bone preparation and reduce variability during the early phase of adoption, supporting lower‐volume or less experienced surgeons in achieving proficiency more quickly [57, 62]. Taken together, these factors may contribute to the observed reduction in implant failure and complications, though the exact mechanism should be in future work.

### 5.5 Operative Time and Cost‐Effectiveness

The longer operative time associated with rUKA is well documented and confirmed by our meta‐analysis, which identified a mean increase of 16.3 min compared with the manual technique [15]. This reflects additional steps such as system setup, registration, and intraoperative planning. While this represents a disadvantage, it may be offset if improved accuracy and lower revision rates translate into better long‐term outcomes. Supporting this, Blyth et al.'s RCT of 139 patients undergoing UKA showed that rUKA became cost‐saving when performed at annual case volumes over 100 in the United Kingdom, including all reintervention costs, finding a saving of £799 per patient undergoing rUKA over a 10‐year period [55]. Although limited by being underpowered and excluding operative time and length of stay, these findings are supported internationally by Moschetti et al., who, using a Markov decision model, also identified rUKA as cost‐effective when annual case volume exceeded 94 procedures [63]. Together, these studies suggest that rUKA can achieve economic viability with sufficient procedural volume. While the technique carries a high upfront cost and may increase operative duration, downstream benefits such as reduced length of stay and lower complication rates have the potential to offset expenses. In the long term, cost savings from rUKA could facilitate higher surgical throughput and support day‐case arthroplasty pathways, helping address elective orthopedic backlogs [60]. Large, sufficiently powered, multicenter cost‐effectiveness studies with subgroup analyses by robot and implant type are needed to more accurately compare the economics of rUKA and mUKA.

### 5.6 Limitations

This systematic review and meta‐analysis has limitations that should be acknowledged. The pooled estimates were associated with high heterogeneity, and while studies were predominantly low‐to‐moderate risk of bias overall, several observational studies were judged to be at serious risk of bias. This may have been driven by numerous confounders. First, patient selection criteria for UKA varied substantially across studies, with differences in age, BMI, degree of deformity. As UKA is offered to healthier, lower‐risk patients in many centers, particularly when performed robotically, this introduces selection bias that may exaggerate the apparent benefits of rUKA. Second, the majority of included studies were conducted in high‐volume specialist centers with significant experience in UKA and early access to robotic technology. This introduces a center bias and may limit generalizability to lower‐volume institutions, where learning curves and resource constraints may alter outcomes. Thirdly, many robotic systems are compatible only with specific implant types, confounding whether observed benefits are attributable to the robotic system or the implant design. Fourth, the median follow‐up duration across studies was approximately 2 years. The lack of long‐term follow‐up precludes comparison of outcomes beyond the early to mid‐term postoperative period. Additionally, most studies were observational in design, with risks of confounding and limited ability to control for surgeon experience, case volume, or institutional protocols. The exclusion of double‐zero event studies may also affect any pooled odds ratios, and the meta‐analyses should be interpreted with caution.

## 6 Conclusion

This systematic review and meta‐analysis provides a comprehensive comparison of rUKA and mUKA. rUKA was associated with significantly lower complication and revision rates, improved functional outcomes, and greater precision in implant positioning. However, these findings should be interpreted cautiously as current evidence is predominantly non‐randomised and may be influenced by confounders such as surgeon experience, center volume, patient selection, implant design, and changes in operative practice over time. Therefore, the observed associations cannot be attributed solely to robotic assistance. Further large, multicenter, and methodologically robust studies are needed to confirm long‐term outcomes, establish UKA‐specific MCID thresholds, and better assess the role of rUKA in clinical practice.

## Author Contributions

**Adrisa Prashar:** methodology, data curation, writing – original draft. **Kapil Sugand:** methodology, investigation, writing – original draft, supervision. **Sree Kanakala:** methodology, writing – original draft, writing – review and editing, formal analysis, investigation, project administration, visualization. **Saran Singh Gill:** writing – review and editing, supervision, conceptualization. **Harjas Singh Shinmar:** writing – original draft, methodology, investigation. **Srikar Reddy Namireddy:** conceptualization, writing – review and editing. **Jennifer Tasong:** methodology, data curation, writing – original draft.

## Funding

Open access funding was provided by the Imperial College Open Access Fund.

## Ethics Statement

The authors have nothing to report.

## Conflicts of Interest

The authors declare no conflicts of interest.

## Supporting information


**Table S1:** Search strategy used for database queries. The Boolean search terms combined synonyms for knee arthroplasty, unicompartmental procedures, and robotic or computer‐assisted techniques to identify all relevant studies comparing robotic‐assisted and manual unicompartmental knee arthroplasty. The search strategy was intentionally kept broad to ensure that no eligible studies were missed.
**Table S2:** Inclusion and exclusion criteria for study selection. Only comparative, English‐language studies of adult patients undergoing robotic‐assisted versus manual unicompartmental knee arthroplasty were included. Non‐comparative, non‐English, or non‐human studies, and those using navigation without a robotic arm, were excluded.
**Table S3:** Risk of bias assessment rubric for randomized studies using the RoB‐2 framework.
**Table S4:** Risk of bias assessment rubric for non‐randomized studies using the ROBINS‐I framework.
**Table S5:** Egger's test *p*‐values. All outcomes did not show statistically significant publication bias.
**Table S6:** Outcome definitions for revision and complication for studies reporting either outcome. Follow‐up duration for each study is reported alongside.
**Figure S1:** Risk of bias assessment for randomized controlled trials using the RoB‐2 tool. (A) Traffic light plot showing domain‐level judgments for each included study across five domains: bias arising from the randomization process, deviations from intended interventions, missing outcome data, measurement of the outcome, and selection of the reported result. (B) Summary plot presenting the proportion of studies rated as “low risk” or having “some concerns” within each bias domain. Overall, most studies demonstrated low risk of bias across domains, with some concerns primarily related to reporting and randomization processes.
**Figure S2:** Risk of bias assessment for non‐randomized studies using the ROBINS‐I tool. (A) Traffic light plot summarizing domain‐level judgments for each included study across seven domains: bias due to confounding, selection of participants, classification of interventions, deviations from intended interventions, missing data, measurement of outcomes, and selection of reported results. (B) Summary plot showing the overall distribution of risk‐of‐bias judgments across all domains. Most studies demonstrated low to moderate risk, with serious risk primarily attributed to confounding and participant selection.
**Figure S3:** Funnel plot assessing publication bias for operative time. Each point represents an individual study plotted by its mean difference and standard error. The symmetrical inverted funnel shape indicates low risk of publication bias, although mild asymmetry on the right side suggests potential small‐study effects favoring longer operative times with robotic‐assisted UKA. Dashed lines denote 95% confidence limits, and shaded regions represent areas of increasing statistical significance.
**Figure S4:** Funnel plot assessing publication bias for length of stay. Each point represents an individual study plotted by its mean difference and standard error. The symmetrical inverted funnel shape indicates low risk of publication bias, with no evidence of small‐study effects. Dashed lines denote 95% confidence limits, and shaded regions represent areas of increasing statistical significance.
**Figure S5:** Funnel plot assessing publication bias for complication rate. Each point represents an individual study plotted by its log odds ratio and standard error. The distribution of studies appears symmetrical around the central line, indicating a low likelihood of publication bias. Dashed lines denote 95% confidence limits, and shaded regions represent areas of increasing statistical significance.
**Figure S6:** Funnel plot assessing publication bias for revision rate. Each point represents an individual study plotted by its log odds ratio and standard error. The distribution of studies is largely symmetrical about the central line, suggesting a low risk of publication bias. Dashed lines indicate 95% confidence limits, and shaded regions represent areas of increasing statistical significance.
**Figure S7:** Funnel plot assessing publication bias for hip‐knee‐ankle angle. Each point represents an individual study plotted by its mean difference and standard error. The distribution of studies appears symmetrical, indicating a low likelihood of publication bias. Dashed lines represent 95% confidence limits, and shaded regions correspond to areas of increasing statistical significance.
**Figure S8:** Funnel plot assessing publication bias for tibial slope angle. Each point represents an individual study plotted by its mean difference and standard error. The plot demonstrates asymmetry, with studies distributed predominantly on the left side of the funnel, suggesting potential small‐study effects or methodological heterogeneity. Dashed lines denote 95% confidence limits, and shaded regions represent areas of increasing statistical significance.
**Figure S9:** Funnel plot assessing publication bias for tibial component angle. Each point represents an individual study plotted by its mean difference and standard error. The distribution of studies appears broadly symmetrical about the central line, indicating a low likelihood of publication bias. Dashed lines denote 95% confidence limits, and shaded regions represent areas of increasing statistical significance.
**Figure S10:** Forest plots comparing odds of complication and revision between robotic‐assisted unicompartmental knee arthroplasty (rUKA) and manual unicompartmental knee arthroplasty (mUKA). Odds ratios of complication and revision are time‐stratified into short, medium and long‐term outcomes.
**Figure 11:** Forest plots comparing patient‐reported outcome measures (PROMs) between robotic‐assisted unicompartmental knee arthroplasty (rUKA) and manual unicompartmental knee arthroplasty (mUKA). PROMs are time‐stratified into short, medium and long‐term outcomes.

## Data Availability

The data that support the findings of this study are available from the corresponding author upon reasonable request.

## References

[1] J. D. Steinmetz , G. T. Culbreth , L. M. Haile , et al., “Global, Regional, and National Burden of Osteoarthritis, 1990 2020 and Projections to 2050: A Systematic Analysis for the Global Burden of Disease Study 2021,” Lancet Rheumatology 5, no. 9 (2023): e508–e522. 10.1016/S2665-9913(23)00163-7 PMC10477960 37675071

[2] R. Duncan , G. Peat , E. Thomas , L. Wood , E. Hay , and P. Croft , “How Do Pain and Function Vary With Compartmental Distribution and Severity of Radiographic Knee Osteoarthritis?,” Rheumatology 47, no. 11 (2008): 1704–1707. 10.1093/rheumatology/ken339 18805874

[3] J. C. Stoddart , O. Dandridge , A. Garner , J. Cobb , and R. J. van Arkel , “The Compartmental Distribution of Knee Osteoarthritis—A Systematic Review and Meta‐Analysis,” Osteoarthritis and Cartilage 29, no. 4 (2021): 445–455. 10.1016/j.joca.2020.10.011 33253887

[4] T. J. Heyse , B. F. El‐Zayat , R. De Corte , et al., “UKA Closely Preserves Natural Knee Kinematics In Vitro,” Knee Surgery, Sports Traumatology, Arthroscopy 22, no. 8 (2014): 1902–1910. 10.1007/s00167-013-2752-0 24213735

[5] A. D. Liddle , H. Pandit , A. Judge , and D. W. Murray , “Optimal Usage of Unicompartmental Knee Arthroplasty,” Bone & Joint Journal 97‐B, no. 1511 (2015): 1506–1511. 10.1302/0301-620X.97B11.35551 26530653

[6] National Joint Registry , 14th Annual Report (2017).

[7] T. W. Hamilton , H. G. Pandit , D. G. Maurer , et al., “Anterior Knee Pain and Evidence of Osteoarthritis of the Patellofemoral Joint Should Not Be Considered Contraindications to Mobile‐Bearing Unicompartmental Knee Arthroplasty: A 15‐Year Follow‐Up,” Bone & Joint Journal 99‐b, no. 5 (2017): 632–639. 10.1302/0301-620X.99B5.BJJ-2016-0695.R2 28455472

[8] O. Robertsson , K. Knutson , S. Lewold , and L. Lidgren , “The Routine of Surgical Management Reduces Failure After Unicompartmental Knee Arthroplasty,” Journal of Bone and Joint Surgery. British Volume 83, no. 1 (2001): 45–49. 10.1302/0301-620x.83b1.10871 11245537

[9] A. D. Liddle , H. Pandit , A. Judge , and D. W. Murray , “Patient‐Reported Outcomes After Total and Unicompartmental Knee Arthroplasty: A Study of 14,076 Matched Patients From the National Joint Registry for England and Wales,” Bone & Joint Journal 97, no. 6 (2015): 793–801. 10.1302/0301-620X.97B6.35155 26033059

[10] J. Blaney , H. Harty , E. Doran , et al., “Five‐Year Clinical and Radiological Outcomes in 257 Consecutive Cementless Oxford Medial Unicompartmental Knee Arthroplasties,” Bone & Joint Journal 99‐b, no. 5 (2017): 623–631. 10.1302/0301-620X.99B5.BJJ-2016-0760.R1 28455471

[11] Q. Naziri , S. A. Burekhovich , P. J. Mixa , et al., “The Trends in Robotic‐Assisted Knee Arthroplasty: A Statewide Database Study,” Journal of Orthopaedics 16, no. 3 (2019): 298–301. 10.1016/j.jor.2019.04.020 PMC6522752 31193224

[12] G. Guild , J. Schwab , B. J. Ross , M. J. McConnell , F. Najafi , and T. L. Bradbury , “Is Robotic‐Assisted Unicompartmental Knee Arthroplasty Compared to Manual Unicompartmental Knee Arthroplasty Associated With Decreased Revision Rates? An Updated Matched Cohort Analysis,” Arthroplasty Today 32 (2025): 101652. 10.1016/j.artd.2025.101652 PMC11932865 40134739

[13] Y. Sun , W. Liu , J. Hou , X. Hu , and W. Zhang , “Does Robotic‐Assisted Unicompartmental Knee Arthroplasty Have Lower Complication and Revision Rates Than the Conventional Procedure? A Systematic Review and Meta‐Analysis,” BMJ Open 11, no. 8 (2021): e044778. 10.1136/bmjopen-2020-044778 PMC8359483 34380715

[14] M. A. Gaudiani , L. T. Samuel , A. F. Kamath , P. M. Courtney , and G. C. Lee , “Robotic‐Assisted Versus Manual Unicompartmental Knee Arthroplasty: Contemporary Systematic Review and Meta‐Analysis of Early Functional Outcomes,” Journal of Knee Surgery 34, no. 10 (2021): 1048–1056. 10.1055/s-0040-1701455 32000264

[15] A. Bensa , A. Sangiorgio , L. Deabate , A. Illuminati , B. Pompa , and G. Filardo , “Robotic‐Assisted Unicompartmental Knee Arthroplasty Improves Functional Outcomes, Complications, and Revisions,” Bone & Joint Open 5, no. 5 (2024): 374–384. 10.1302/2633-1462.55.BJO-2024-0030.R1 PMC11061807 38690670

[16] B. Z. Chin , S. S. H. Tan , K. C. X. Chua , G. R. Budiono , N. L. Syn , and G. K. O'Neill , “Robot‐Assisted Versus Conventional Total and Unicompartmental Knee Arthroplasty: A Meta‐Analysis of Radiological and Functional Outcomes,” Journal of Knee Surgery 34, no. 10 (2021): 1064–1075. 10.1055/s-0040-1701440 32185785

[17] F. Zhang , H. Li , Z. Ba , C. Bo , and K. Li , “Robotic Arm‐Assisted vs Conventional Unicompartmental Knee Arthroplasty: A Meta‐Analysis of the Effects on Clinical Outcomes,” Medicine 98, no. 35 (2019): e16968. 10.1097/MD.0000000000016968 PMC6736485 31464939

[18] M. J. Page , J. E. McKenzie , P. M. Bossuyt , et al., “The PRISMA 2020 Statement: An Updated Guideline for Reporting Systematic Reviews,” BMJ 372 (2021): n71. 10.1136/bmj.n71 PMC8005924 33782057

[19] J. A. C. Sterne , M. A. Hernán , B. C. Reeves , et al., “ROBINS‐I: A Tool for Assessing Risk of Bias in Non‐Randomised Studies of Interventions,” BMJ 355 (2016): i4919. 10.1136/bmj.i4919 PMC5062054 27733354

[20] J. A. C. Sterne , J. Savović , M. J. Page , et al., “RoB 2: A Revised Tool for Assessing Risk of Bias in Randomised Trials,” BMJ 366 (2019): l4898. 10.1136/bmj.l4898 31462531

[21] I. C. Jeremy Howick , P. Glasziou , T. Greenhalgh , et al., The 2011 Oxford CEBM Levels of Evidence (Introductory Document) (2011).

[22] S. Balduzzi , G. Rücker , and G. Schwarzer , “How to Perform a Meta‐Analysis With R: A Practical Tutorial,” Evidence‐Based Mental Health 22, no. 4 (2019): 153–160. 10.1136/ebmental-2019-300117 PMC10231495 31563865

[23] J. Cheng , E. Pullenayegum , J. K. Marshall , A. Iorio , and L. Thabane , “Impact of Including or Excluding Both‐Armed Zero‐Event Studies on Using Standard Meta‐Analysis Methods for Rare Event Outcome: A Simulation Study,” BMJ Open 6, no. 8 (2016): e010983. 10.1136/bmjopen-2015-010983 PMC5013416 27531725

[24] X. Wan , W. Wang , J. Liu , and T. Tong , “Estimating the Sample Mean and Standard Deviation From the Sample Size, Median, Range and/or Interquartile Range,” BMC Medical Research Methodology 14 (2014): 135. 10.1186/1471-2288-14-135 PMC4383202 25524443

[25] M. Campbell , J. E. McKenzie , A. Sowden , et al., “Synthesis Without Meta‐Analysis (SWiM) in Systematic Reviews: Reporting Guideline,” BMJ 368 (2020): l6890. 10.1136/bmj.l6890 PMC7190266 31948937

[26] C. Tan , S. Shih , V. Ravichandra , E. Quah , and R. Kunnasegaran , “Clinical Outcome Scores Post Medial Unicompartmental Knee Arthroplasty: A Comparison of the MAKO Robotic Arm Versus the Oxford Conventional Approach,” Malaysian Orthopaedic Journal 19, no. 1 (2025): 3–10. 10.5704/MOJ.2503.002 PMC12022716 40291971

[27] V. Rossi , C. Foissey , A. Fontalis , et al., “Robotic Assistance in Simultaneous Bilateral Medial Unicompartmental Knee Arthroplasty: A Retrospective Cohort Study of 126 Knees Demonstrating Enhanced Radiographic Accuracy and Comparable Safety to Conventional Methods,” Arthroplast Today 31 (2025): 101594. 10.1016/j.artd.2024.101594 PMC11788786 39902168

[28] R. Sun , W. Yilihamujiang , A. Aierxiding , et al., “China‐Made Robot‐Assisted Unicompartmental Knee Arthroplasty Can Enhance the Precision of Prosthesis Placement and Yield Better Short‐Term Efficacy,” Journal of Orthopaedic Surgery (Hong Kong) 32, no. 3 (2024): 10225536241298040. 10.1177/10225536241298040 39644165

[29] P. L. Lim , Z. Sayeed , M. R. Gonzalez , C. M. Melnic , and H. S. Bedair , “Comparison of Time and Rate of Achieving Minimal Clinically Important Difference: Robotic Versus Manual Unicompartmental Knee Arthroplasty,” Journal of the American Academy of Orthopaedic Surgeons 33, no. 5 (2025): 231–241. 10.5435/JAAOS-D-24-00380 39083490

[30] W. H. Lau , W. K. T. Liu , K. Y. Chiu , et al., “Reducing Edge Loading and Alignment Outliers With Image‐Free Robotic‐Assisted Unicompartmental Knee Arthroplasty: A Case Controlled Study,” Art 6, no. 1 (2024): 33. 10.1186/s42836-024-00259-x PMC11151636 38835099

[31] S. C. Kwon , H. J. Jung , J. H. Lee , J. T. Hyun , J. H. Hwang , and J. I. Kim , “Robotic‐Assisted Medial Unicompartmental Knee Arthroplasty Restored Prearthritic Alignment and Led to Superior Functional Outcomes Compared With Conventional Techniques,” Knee Surgery, Sports Traumatology, Arthroscopy 33, no. 1 (2025): 265–273. 10.1002/ksa.12278 38796719

[32] M. H. Y. Yeung , H. Fu , A. Cheung , et al., “Robotic Arm‐Assisted Unicondylar Knee Arthroplasty Resulted in Superior Radiological Accuracy: A Propensity Score‐Matched Analysis,” Art 5, no. 1 (2023): 55. 10.1186/s42836-023-00210-6 PMC10621242 37915082

[33] C. Foissey , C. Batailler , A. Vahabi , A. Fontalis , E. Servien , and S. Lustig , “Better Accuracy and Implant Survival in Medial Imageless Robotic‐Assisted Unicompartmental Knee Arthroplasty Compared to Conventional Unicompartmental Knee Arthroplasty: Two‐ to Eleven‐Year Follow‐Up of Three Hundred Fifty‐Six Consecutive Knees,” International Orthopaedics 47, no. 2 (2023): 533–541. 10.1007/s00264-022-05640-6 36434294

[34] G. Maritan , G. Franceschi , R. Nardacchione , et al., “Similar Survivorship at the 5‐Year Follow‐Up Comparing Robotic‐Assisted and Conventional Lateral Unicompartmental Knee Arthroplasty,” Knee Surgery, Sports Traumatology, Arthroscopy 31, no. 3 (2023): 1063–1071. 10.1007/s00167-022-07218-6 PMC9958141 36374325

[35] M. P. Crizer , A. Haffar , A. Battenberg , M. McGrath , R. Sutton , and J. H. Lonner , “Robotic Assistance in Unicompartmental Knee Arthroplasty Results in Superior Early Functional Recovery and Is More Likely to Meet Patient Expectations,” Advances in Orthopedics 2021 (2021): 4770960. 10.1155/2021/4770960 PMC8298171 34336292

[36] M. Banger , J. Doonan , P. Rowe , B. Jones , A. MacLean , and M. J. B. Blyth , “Robotic Arm‐Assisted Versus Conventional Medial Unicompartmental Knee Arthroplasty: Five‐Year Clinical Outcomes of a Randomized Controlled Trial,” Bone & Joint Journal 103, no. 6 (2021): 1088–1095. 10.1302/0301-620X.103B6.BJJ-2020-1355.R2 PMC8153511 34058870

[37] R. Negrín , J. Duboy , M. Iñiguez , et al., “Robotic‐Assisted vs Conventional Surgery in Medial Unicompartmental Knee Arthroplasty: A Clinical and Radiological Study,” Knee Surgery & Related Research 33, no. 1 (2021): 5. 10.1186/s43019-021-00087-2 PMC7879692 33579393

[38] J. Thilak , M. Thadi , P. P. Mane , A. Sharma , V. Mohan , and B. C. Babu , “Accuracy of Tibial Component Positioning in the Robotic Arm Assisted Versus Conventional Unicompartmental Knee Arthroplasty,” Journal of Orthopaedics 22 (2020): 367–371. 10.1016/j.jor.2020.08.022 PMC7486415 32952328

[39] G. Mergenthaler , C. Batailler , T. Lording , E. Servien , and S. Lustig , “Is Robotic‐Assisted Unicompartmental Knee Arthroplasty a Safe Procedure? A Case Control Study,” Knee Surgery, Sports Traumatology, Arthroscopy 29, no. 3 (2021): 931–938. 10.1007/s00167-020-06051-z 32390119

[40] J. P. St Mart , R. N. de Steiger , A. Cuthbert , and W. Donnelly , “The Three‐Year Survivorship of Robotically Assisted Versus Non‐Robotically Assisted Unicompartmental Knee Arthroplasty,” Bone & Joint Journal 102 (2020): 319–328. 10.1302/0301-620X.102B3.BJJ-2019-0713.R1 32114810

[41] K. K. Park , C. D. Han , I. H. Yang , W. S. Lee , J. H. Han , and H. M. Kwon , “Robot‐Assisted Unicompartmental Knee Arthroplasty Can Reduce Radiologic Outliers Compared to Conventional Techniques,” PLoS One 14, no. 12 (2019): e0225941. 10.1371/journal.pone.0225941 PMC6890211 31794587

[42] R. M. Vakharia , N. Sodhi , W. B. Cohen‐Levy , A. M. Vakharia , M. A. Mont , and M. W. Roche , “Comparison of Patient Demographics and Utilization Trends of Robotic‐Assisted and Non‐Robotic‐Assisted Unicompartmental Knee Arthroplasty,” Journal of Knee Surgery 34, no. 6 (2021): 621–627. 10.1055/s-0039-1698769 31639851

[43] C. L. Cool , K. A. Needham , A. Khlopas , and M. A. Mont , “Revision Analysis of Robotic Arm‐Assisted and Manual Unicompartmental Knee Arthroplasty,” Journal of Arthroplasty 34, no. 5 (2019): 926–931. 10.1016/j.arth.2019.01.018 31010509

[44] B. Kayani , S. Konan , J. Tahmassebi , F. E. Rowan , and F. S. Haddad , “An Assessment of Early Functional Rehabilitation and Hospital Discharge in Conventional Versus Robotic‐Arm Assisted Unicompartmental Knee Arthroplasty: A Prospective Cohort Study,” Bone & Joint Journal 101, no. 1 (2019): 24–33. 10.1302/0301-620X.101B1.BJJ-2018-0564.R2 30601042

[45] J. Wong , T. Murtaugh , A. Lakra , H. J. Cooper , R. P. Shah , and J. A. Geller , “Robotic‐Assisted Unicompartmental Knee Replacement Offers no Early Advantage Over Conventional Unicompartmental Knee Replacement,” Knee Surgery, Sports Traumatology, Arthroscopy 27, no. 7 (2019): 2303–2308. 10.1007/s00167-019-05386-6 30747237

[46] R. Canetti , C. Batailler , C. Bankhead , P. Neyret , E. Servien , and S. Lustig , “Faster Return to Sport After Robotic‐Assisted Lateral Unicompartmental Knee Arthroplasty: A Comparative Study,” Archives of Orthopaedic and Trauma Surgery 138, no. 12 (2018): 1765–1771. 10.1007/s00402-018-3042-6 30242566

[47] C. Batailler , N. White , F. M. Ranaldi , P. Neyret , E. Servien , and S. Lustig , “Improved Implant Position and Lower Revision Rate With Robotic‐Assisted Unicompartmental Knee Arthroplasty,” Knee Surgery, Sports Traumatology, Arthroscopy 27, no. 4 (2019): 1232–1240. 10.1007/s00167-018-5081-5 30066017

[48] A. Gilmour , A. D. MacLean , P. J. Rowe , et al., “Robotic‐Arm‐Assisted Versus Conventional Unicompartmental Knee Arthroplasty. The 2‐Year Clinical Outcomes of a Randomized Controlled Trial,” Journal of Arthroplasty 33, no. 7S (2018): S109–S115. 10.1016/j.arth.2018.02.050 29627257

[49] M. J. G. Blyth , I. Anthony , P. Rowe , M. S. Banger , A. MacLean , and B. Jones , “Robotic Arm‐Assisted Versus Conventional Unicompartmental Knee Arthroplasty: Exploratory Secondary Analysis of a Randomised Controlled Trial,” Bone Joint Research 6, no. 11 (2017): 631–639. 10.1302/2046-3758.611.BJR-2017-0060.R1 PMC5717071 29162608

[50] K. P. MacCallum , J. R. Danoff , and J. A. Geller , “Tibial Baseplate Positioning in Robotic‐Assisted and Conventional Unicompartmental Knee Arthroplasty,” European Journal of Orthopaedic Surgery & Traumatology 26, no. 1 (2016): 93–98. 10.1007/s00590-015-1708-0 26441329

[51] D. C. Hansen , S. K. Kusuma , R. M. Palmer , and K. B. Harris , “Robotic Guidance Does Not Improve Component Position or Short‐Term Outcome in Medial Unicompartmental Knee Arthroplasty,” Journal of Arthroplasty 29, no. 9 (2014): 1784–1789. 10.1016/j.arth.2014.04.012 24851792

[52] J. H. Lonner , T. K. John , and M. A. Conditt , “Robotic Arm‐Assisted UKA Improves Tibial Component Alignment: A Pilot Study,” Clinical Orthopaedics and Related Research 468, no. 1 (2010): 141–146. 10.1007/s11999-009-0977-5 PMC2795844 19593669

[53] J. Cobb , J. Henckel , P. Gomes , et al., “Hands‐On Robotic Unicompartmental Knee Replacement: A Prospective, Randomised Controlled Study of the Acrobot System,” Journal of Bone and Joint Surgery 88, no. 2 (2006): 188–197. 10.1302/0301-620X.88B2.17220 16434522

[54] A. V. Strait , J. W. Lee , R. Supra , et al., “Comparing Clinical and Patient‐Reported Outcomes Between Robot‐Assisted and Conventional Unicompartmental Knee Arthroplasty,” Current Orthopaedic Practice 36, no. 1 (2024): e1281.

[55] M. J. G. Blyth , X. Y. Choo , J. Doonan , A. MacLean , B. G. Jones , and N. D. Clement , “Robotic Arm‐Assisted Medial Compartment Knee Arthroplasty Is a Cost‐Effective Intervention at Ten‐Year Follow‐Up: A Cost‐Effectiveness Analysis From s Randomized Controlled Trial,” Bone & Joint Journal 107, no. 1 (2025): 72–80. 10.1302/0301-620X.107B1.BJJ-2024-0245.R2 39740684

[56] C. Wu , N. Fukui , Y. K. Lin , et al., “Comparison of Robotic and Conventional Unicompartmental Knee Arthroplasty Outcomes in Patients With Osteoarthritis: A Retrospective Cohort Study,” Journal of Clinical Medicine 11, no. 1 (2022): 220. 10.3390/jcm11010220 PMC8745819 35011960

[57] B. Kayani , S. Konan , J. R. T. Pietrzak , S. S. Huq , J. Tahmassebi , and F. S. Haddad , “The Learning Curve Associated With Robotic‐Arm Assisted Unicompartmental Knee Arthroplasty,” Bone & Joint Journal 100, no. 8 (2018): 1033–1042. 10.1302/0301-620X.100B8.BJJ-2018-0040.R1 30062950

[58] S. W. Bell , I. Anthony , B. Jones , A. MacLean , P. Rowe , and M. Blyth , “Improved Accuracy of Component Positioning With Robotic‐Assisted Unicompartmental Knee Arthroplasty: Data From a Prospective, Randomized Controlled Study,” Journal of Bone and Joint Surgery 98, no. 8 (2016): 627–635. 10.2106/JBJS.15.00664 27098321

[59] W. C. Lee , Y. H. Kwan , H. C. Chong , and S. J. Yeo , “The Minimal Clinically Important Difference for Knee Society Clinical Rating System After Total Knee Arthroplasty for Primary Osteoarthritis,” Knee Surgery, Sports Traumatology, Arthroscopy 25, no. 11 (2017): 3354–3359. 10.1007/s00167-016-4208-9 27324635

[60] B. Kayani and F. S. Haddad , “Robotic Unicompartmental Knee Arthroplasty: Current Challenges and Future Perspectives,” Bone & Joint Research 8, no. 6 (2019): 228–231. 10.1302/2046-3758.86.BJR-2019-0037 PMC6609866 31346450

[61] R. Negrín , J. Duboy , N. O. Reyes , et al., “Robotic‐Assisted Unicompartmental Knee Arthroplasty Optimizes Joint Line Restitution Better Than Conventional Surgery,” Journal of Experimental Orthopaedics 7, no. 1 (2020): 94. 10.1186/s40634-020-00309-8 PMC7701039 33251551

[62] B. Kayani , S. Konan , J. R. T. Pietrzak , S. S. Huq , J. Tahmassebi , and F. S. Haddad , “The Learning Curve Associated With Robotic‐Arm Assisted Unicompartmental Knee Arthroplasty: A Prospective Cohort Study,” Bone & Joint Journal 100, no. 8 (2018): 1033–1042. 10.1302/0301-620X.100B8.BJJ-2018-0040.R1 30062950

[63] W. E. Moschetti , J. F. Konopka , H. E. Rubash , and J. W. Genuario , “Can Robot‐Assisted Unicompartmental Knee Arthroplasty be Cost‐Effective? A Markov Decision Analysis,” Journal of Arthroplasty 31, no. 4 (2016): 759–765. 10.1016/j.arth.2015.10.018 26706836

