# Supplementary material for: Robotic Assisted Versus Manual Unicompartmental Knee Arthroplasty: A Systematic Review and Meta‐Analysis
Source: Orthop Surg. 2026 Oct 22:10.1111/os.70424. Online ahead of print. doi: 10.1111/os.70424 (PMC13687996; doi:10.1111/os.70424)
Supplement: Supplementary file 1 [file OS-9999-0-s001.docx]

**Supplementary Tables**

*Table 1:* Search Strategy

*Table 2:* Inclusion & Exclusion Criteria

*Table 3:* Randomized Study Risk of Bias Assessment Rubric

*Table 4:* Non-Randomized Study Risk of Bias Assessment Rubric

*Table 5:* Egger’s Test Results

*Table 6:* Complication and Revision Rate Outcome Definition

(“Knee arthroplasty” OR “Knee replacement” OR "knee”)

AND

(“Unicompartmental” OR “UKA”)

AND

(“Robotic-assisted” OR “Robotic-arm” OR “Robotic” OR “Computer-assisted”)

**Supplementary Table 1: Search strategy used for database queries.** The Boolean search terms combined synonyms for knee arthroplasty, unicompartmental procedures, and robotic or computer-assisted techniques to identify all relevant studies comparing robotic-assisted and manual unicompartmental knee arthroplasty. The search strategy was intentionally kept broad to ensure that no eligible studies were missed.

| **Inclusion criteria** | **Exclusion criteria** |
| --- | --- |
| - Published in the English language - Peer-reviewed journals - Comparative studies with both mUKA and rUKA cohorts - Adult human patients undergoing UKA - Studies evaluating clinical (complication rate, revision rate), perioperative (operative time, length of stay), radiographic (HKAA, TSA, TCA) and patient-reported outcomes | - All non-English languages - Commentaries, case reports, narrative reviews, letters to editors, books - Any animal studies and lab-based studies - Studies on children and adolescents (<18 years) - Navigation-assisted UKA without robotic arm component |

**Supplementary Table 2:** Inclusion and exclusion criteria for study selection. Only comparative, English-language studies of adult patients undergoing robotic-assisted versus manual unicompartmental knee arthroplasty were included. Non-comparative, non-English, or non-human studies, and those using navigation without a robotic arm, were excluded.

| **Domain** | **Low risk** | **Some concerns** | **High risk** |
| --- | --- | --- | --- |
| **D1: Randomisation** | Adequate randomisation and concealment | Methods unclear or minor imbalance | Non-random or unconcealed allocation |
| **D2: Deviations** | No important deviations; appropriate analysis | Adherence or analysis incompletely reported | Large deviations or inappropriate analysis |
| **D3: Missing data** | Minimal or unlikely to bias results | Missingness insufficiently explained | Substantial missing data |
| **D4: Outcome measurement** | Valid, consistent, and objective/blinded | Blinding unclear or some subjectivity | Measurement differed or was likely biased |
| **D5: Selective reporting** | Consistent with protocol or stated methods | No protocol or analysis plan available | Evidence of selective outcome or analysis reporting |
| **Overall** | Low risk in all domains | Some concerns in ≥1 domain, none high | High risk in ≥1 domain |

**Supplementary Table 3: Risk of bias assessment rubric for randomized studies using the RoB-2 framework.**

| **Domain** | **Low risk** | **Moderate risk** | **Serious/Critical risk** |
| --- | --- | --- | --- |
| **D1: Confounding** | Important confounders adequately controlled | Some residual or unclear confounding | Major confounders unmeasured or uncontrolled |
| **D2: Participant selection** | Selection unrelated to intervention and outcome | Selection process incompletely described | Selection likely related to intervention and outcome |
| **D3: Intervention classification** | Intervention clearly and correctly classified | Minor uncertainty in classification | Substantial or differential misclassification |
| **D4: Deviations from intervention** | No important deviations, appropriate analysis | Deviations or adherence incompletely reported | Important deviations likely to affect outcomes |
| **D5: Missing data** | Minimal or unlikely to bias results | Missingness insufficiently explained | Substantial or differential missing data |
| **D6: Outcome measurement** | Valid and comparable measurement between groups | Some subjectivity or unclear blinding | Measurement differed or was likely biased |
| **D7: Selective reporting** | Outcomes and analyses reported as planned | No protocol or analysis plan available | Evidence of selective reporting |
| **Overall** | Low risk in all domains | No serious/critical domains, but ≥1 moderate | Serious/critical risk in ≥1 domain |

**Supplementary Table 4: Risk of bias assessment rubric for non-randomized studies using the ROBINS-I framework.**

| **Outcome** | **Bias Estimate** | **p-value** |
| --- | --- | --- |
| Complication Rate Odds Ratio | -0.49 | 0.13 |
| Revision Rate Odds Ratio | 0.61 | 0.43 |
| Hip Knee Ankle Angle Mean Difference | 2.30 | 0.49 |
| Tibial Component Angle Mean Difference | 0.07 | 0.99 |
| Tibial Slope Mean Difference | 3.57 | 0.21 |
| Length of Stay Mean Difference | 2.47 | 0.59 |
| Operative Time Mean Difference | 0.72 | 0.77 |

**Supplementary Table 5: Egger’s test p-values.** All outcomes did not show statistically significant publication bias.

| **Study** | **Complication Definition** | **Revision Definition** | **Complication Follow-Up Window** |
| --- | --- | --- | --- |
| Yeung 2023 |  | Reported but not defined. Reported revisions were for tibial component loosening. |  |
| Wong 2019 |  | Any revision surgery. |  |
| Strait 2024 | Infection, DVT, PE, arthrofibrosis requiring manipulation, and periprosthetic fracture. | Cumulative revision surgery identified from surgical logs and electronic health records. | 90 days |
| St Mart 2020 |  | Time to first revision for any cause - removal, replacement, or addition of any prosthetic component. |  |
| Rossi 2025 | Any medical complication | Revision to total knee arthroplasty during follow-up. | Within 60 days |
| Mergenthaler 2021 | Any robotic system related complication | Revision with implant removal. | 2 months, 6 months, 1 year, and latest follow-up |
| Maritan 2023 |  | Implant failure due to PJI, periprosthetic fracture, aseptic loosening, or revision to TKA for medial or patellar cartilage deterioration. |  |
| MacCallum 2016 |  | Failure requiring revision surgery for mechanical causes. |  |
| Lonner 2010 |  | Reported but not defined. |  |
| Lim 2025 | DVT, PE, pneumonia, cellulitis, aseptic loosening, stiffness, and PJI. | Reported but not defined. | 90 days |
| Lau 2024 | Reported events included unexplained pain and aseptic loosening, but not defined. | Reported but not defined. Reported revisions were conversion to TKA for unexplained pain or aseptic loosening. | Mean 3 years; minimum 8 months |
| Guild 2025 | Any medical or prosthesis-related adverse event | Aseptic revision included revision UKA or conversion to TKA involving femoral and/or tibial components or liner exchange. | Medical complications: 90 days; prosthesis-related complications: 1 and 2 years |
| Foissey 2023 | Reported but not defined | Any surgical intervention after the index procedure, including implant removal, revision to TKA for infection, loosening or OA progression, or implantation of a lateral UKA or patellofemoral prosthesis in the same knee. | 2 months, 6 months, 1 year, and latest follow-up |
| Cool 2019 |  | Hospital admission for a surgical knee revision identified using specified ICD-9/10 procedure codes. |  |
| Batailler 2019 | Reported but not defined | Revision to another UKA or to TKA. Reoperations without prosthesis revision were reported separately. |  |
| Banger 2021 | Reported but not defined | Revision was explantation of a primary component, excluding bearing exchange. All-cause reintervention included any further surgical procedure on the knee. | 3 months, 1 year, 2 years, and 5 years |
| Blyth 2017 | Reported but not defined | Requirement for revision surgery was recorded; no formal revision definition was provided. | 3 months and 1 year |
| Wu 2022 | Reported but not defined |  | 6 months, 1 year, and 2 years |
| Tan 2025 | Postoperative complications reported included DVT, periprosthetic fracture, mechanical failure, and infection | Reported but not defined | Up to 2 years |
| Negrín 2021 | Any postoperative complication identified from the clinical record |  | 6 months |
| Kayani 2018 | Any postoperative complication and its treatment identified at outpatient review; no prespecified event list was provided. |  | 30 days |
| Kayani 2019 | Complications occurring after surgery were prospectively recorded; no prespecified event list was provided. |  | Within 90 days |
| Hansen 2014 | Postoperative adverse events identified by medical-record review, including deep infection, cellulitis, and persistent medial-sided pain. | Any revision surgery. | Minimum 24 months |
| Cobb 2006 | All adverse events, including device- and procedure-related complications. |  | 6 and 18 weeks |
| Canetti 2018 | Reported but not defined. | Conversion to TKA or replacement with another UKA. | Mean 34.4 months (rUKA) and 39.3 months (mUKA) |
| Blyth 2025 | Reported but not defined. | All-cause revision was conversion/revision to TKA; aseptic revision excluded infection-related procedures. | Up to 10 years |

**Supplementary Table 6: Outcome definitions for revision and complication for studies reporting either outcome.** Follow-up duration for each study is reported alongside.

**Supplementary Figures**

*Supplementary Figure 1:* RoB-2 assessment of RCTs

*Supplementary Figure 2:* ROBINS-I assessment of non-randomised studies

*Supplementary Figure 3:* Operative Time Funnel Plot

*Supplementary Figure 4:* Length of Stay Funnel Plot

*Supplementary Figure 5:* Complication Rate Funnel Plot

*Supplementary Figure 6:* Revision Rate Funnel Plot

*Supplementary Figure 7:* Hip-Knee-Ankle Angle Funnel Plot

*Supplementary Figure 8:* Tibial Slope Angle Funnel Plot

*Supplementary Figure 9:* Tibial Component Angle Funnel Plot

*Supplementary Figure 10:* Time-Stratified Complication and Revision Rate Forest Plots

*Supplementary Figure 11:* Time-Stratified Patient Reported Outcomes Measure Forest Plots

B

A

**Supplementary Figure 1: Risk of bias assessment for randomized controlled trials using the RoB-2 tool.** **(A) Traffic light plot** showing domain-level judgments for each included study across five domains: bias arising from the randomization process, deviations from intended interventions, missing outcome data, measurement of the outcome, and selection of the reported result. **(B) Summary plot** presenting the proportion of studies rated as “low risk” or having “some concerns” within each bias domain. Overall, most studies demonstrated low risk of bias across domains, with some concerns primarily related to reporting and randomization processes.

B

A

**Supplementary Figure 2: Risk of bias assessment for non-randomized studies using the ROBINS-I tool. (A) Traffic light plot** summarizing domain-level judgments for each included study across seven domains: bias due to confounding, selection of participants, classification of interventions, deviations from intended interventions, missing data, measurement of outcomes, and selection of reported results**. (B) Summary plot** showing the overall distribution of risk-of-bias judgments across all domains. Most studies demonstrated low to moderate risk, with serious risk primarily attributed to confounding and participant selection.

**Supplementary Figure 3: Funnel plot assessing publication bias for operative time.** Each point represents an individual study plotted by its mean difference and standard error. The symmetrical inverted funnel shape indicates low risk of publication bias, although mild asymmetry on the right side suggests potential small-study effects favouring longer operative times with robotic-assisted UKA. Dashed lines denote 95% confidence limits, and shaded regions represent areas of increasing statistical significance.

**Supplementary Figure 4: Funnel plot assessing publication bias for length of stay**. Each point represents an individual study plotted by its mean difference and standard error. The symmetrical inverted funnel shape indicates low risk of publication bias, with no evidence of small-study effects. Dashed lines denote 95% confidence limits, and shaded regions represent areas of increasing statistical significance.

**Supplementary Figure 5: Funnel plot assessing publication bias for complication rate.** Each point represents an individual study plotted by its log odds ratio and standard error. The distribution of studies appears symmetrical around the central line, indicating a low likelihood of publication bias. Dashed lines denote 95% confidence limits, and shaded regions represent areas of increasing statistical significance.

**Supplementary Figure 6: Funnel plot assessing publication bias for revision rate.** Each point represents an individual study plotted by its log odds ratio and standard error. The distribution of studies is largely symmetrical about the central line, suggesting a low risk of publication bias. Dashed lines indicate 95% confidence limits, and shaded regions represent areas of increasing statistical significance.

**Supplementary Figure 7: Funnel plot assessing publication bias for hip-knee-ankle angle.** Each point represents an individual study plotted by its mean difference and standard error. The distribution of studies appears symmetrical, indicating a low likelihood of publication bias. Dashed lines represent 95% confidence limits, and shaded regions correspond to areas of increasing statistical significance.

**Supplementary Figure 8: Funnel plot assessing publication bias for tibial slope angle.** Each point represents an individual study plotted by its mean difference and standard error. The plot demonstrates asymmetry, with studies distributed predominantly on the left side of the funnel, suggesting potential small-study effects or methodological heterogeneity. Dashed lines denote 95% confidence limits, and shaded regions represent areas of increasing statistical significance.

**Supplementary Figure 9: Funnel plot assessing publication bias for tibial component angle.** Each point represents an individual study plotted by its mean difference and standard error. The distribution of studies appears broadly symmetrical about the central line, indicating a low likelihood of publication bias. Dashed lines denote 95% confidence limits, and shaded regions represent areas of increasing statistical significance.

**D**

**A**

**Supplementary Figure 10: Forest plots comparing odds of complication and revision between robotic-assisted unicompartmental knee arthroplasty (rUKA) and manual unicompartmental knee arthroplasty (mUKA).** Odds ratios of complication and revision are time-stratified into short, medium and long-term outcomes.

**E**

**C**

**B**

**F**

**A**

**B**

**A**

**A**

**A**

**A**

**A**

**A**

**Supplementary Figure 11: Forest plots comparing patient-reported outcome measures (PROMs) between robotic-assisted unicompartmental knee arthroplasty (rUKA) and manual unicompartmental knee arthroplasty (mUKA).** PROMs are time-stratified into short, medium and long-term outcomes.

**I**

**H**

**G**

**E**

**D**

**C**
